# Topographical Heterogeneity of Alzheimer’s Disease Based on MR Imaging, Tau PET, and Amyloid PET

**DOI:** 10.3389/fnagi.2019.00211

**Published:** 2019-08-20

**Authors:** Seun Jeon, Jae Myeong Kang, Seongho Seo, Hye Jin Jeong, Thomas Funck, Sang-Yoon Lee, Kee Hyung Park, Yeong-Bae Lee, Byeong Kil Yeon, Tatsuo Ido, Nobuyuki Okamura, Alan C. Evans, Duk L. Na, Young Noh

**Affiliations:** ^1^McGill Centre for Integrative Neuroscience, Montreal Neurological Institute, McGill University, Montreal, QC, Canada; ^2^Department of Psychiatry, Gil Medical Center, Gachon University College of Medicine, Incheon, South Korea; ^3^Department of Neuroscience, Gachon University College of Medicine, Incheon, South Korea; ^4^Neuroscience Research Institute, Gachon University, Incheon, South Korea; ^5^Department of Neurology, Gil Medical Center, Gachon University College of Medicine, Incheon, South Korea; ^6^Division of Pharmacology, Faculty of Medicine, Tohoku Medical and Pharmaceutical University, Sendai, Japan; ^7^Department of Neurology, Samsung Medical Center, Sungkyunkwan University School of Medicine, Seoul, South Korea; ^8^Neuroscience Center, Samsung Medical Center, Seoul, South Korea; ^9^Department of Health Science and Technology, GAIHST, Gachon University, Incheon, South Korea

**Keywords:** Alzheimer’s disease, cluster analysis, tau, amyloid, cortical thickness, positron emission tomography, magnetic resonance imaging

## Abstract

Alzheimer’s disease (AD) patients are known to have heterogeneous clinical presentation and pathologic patterns. We hypothesize that AD dementia can be categorized into subtypes based on multimodal imaging biomarkers such as magnetic resonance imaging (MRI), tau positron emission tomography (PET), and amyloid PET. We collected 3T MRI, ^18^F-THK5351 PET, and ^18^F-flutemetamol (FLUTE) PET data from 83 patients with AD dementia [Clinical Dementia Rating (CDR) ≤1] and 60 normal controls (NC), and applied surface-based analyses to measure cortical thickness, THK5351 standardized uptake value ratio (SUVR) and FLUTE SUVR for each participant. For the patient group, we performed an agglomerative hierarchical clustering analysis using the three multimodal imaging features on the vertices (*n* = 3 × 79,950). The identified AD subtypes were compared to NC using general linear models adjusting for age, sex, and years of education. We mapped the effect size within significant cortical regions reaching a corrected *p*-vertex <0.05 (random field theory). Our surface-based multimodal framework has revealed three distinct subtypes among AD patients: medial temporal-dominant subtype (MT, *n* = 44), parietal-dominant subtype (P, *n* = 19), and diffuse atrophy subtype (D, *n* = 20). The topography of cortical atrophy and THK5351 retention differentiates between the three subtypes. In the case of FLUTE, three subtypes did not show distinct topographical differences, although cortical composite retention was significantly higher in the P type than in the MT type. These three subtypes also differed in demographic and clinical features. In conclusion, AD patients may be clustered into three subtypes with distinct topographical features of cortical atrophy and tau deposition, although amyloid deposition may not differ across the subtypes in terms of topography.

## Introduction

Alzheimer’s disease (AD) is a neurodegenerative disease characterized by histopathologic lesions of amyloid plaques and neurofibrillary tangles composed of the β-amyloid (Aβ) protein and paired helical filaments of hyperphosphorylated tau protein, respectively (Hyman and Trojanowski, 1997). These two neuropathologic hallmarks of AD are known to have typical spreading patterns. The Aβ accumulation has been known to progress from the neocortex to the brainstem (Thal et al., 2002) and neurofibrillary tangle pathology initially appears in the transentorhinal region and spreads to the limbic area, association cortices, and finally the primary cortices (Braak and Braak, 1991). Structural lesions including hippocampal and medial temporal cortical atrophy are also known to be specific to AD and can be used to screen and track the progression of AD (Scheltens et al., 1992; Frisoni et al., 2008). An A/T/N classification has been accepted for the description of multidomain biomarker findings for amyloid, tau, and neurodegeneration (Jack et al., 2016).

Previous studies have suggested anatomical and neuropathologic heterogeneity in AD. A postmortem study has found that neurofibrillary tangles can be a determinant of variability in AD (Murray et al., 2011). In terms of atrophic patterns, a voxel-based morphometry study has classified AD into four subgroups according to the regional atrophy (Shiino et al., 2006). It was reported that patterns of atrophy on magnetic resonance imaging (MRI) had three subtypes, which concomitantly correlated with pathological subtypes (Whitwell et al., 2012). Our group has also previously classified a large group of early AD dementia into three subtypes according to the regional cortical thickness (Noh et al., 2014). A recent tau positron emission tomography (PET) study using cluster analysis has reported variability of tau PET uptake in AD (Whitwell et al., 2018). In addition, these AD subtypes showed distinct clinical and demographic characteristics (Murray et al., 2011; Noh et al., 2014; Whitwell et al., 2018) and long-term disease progression (Na et al., 2016). Further investigation of the subtypes of AD dementia may facilitate a deeper understanding of its characteristics and progression.

With the development of PET tracers, researchers now can observe tau pathologies *in vivo*. Recent studies have shown that tau PET tracers such as ^18^F-THK5351 and ^18^F-AV-1451 significantly differentiate AD patients from old adults with normal cognition, reflect disease progression in AD, and correlates with neurofibrillary tangle retention (Cho et al., 2016; Schwarz et al., 2016; Schöll et al., 2016; Kang et al., 2017). Tau PET tracers also have been reported to bind to non-AD tauopathies, hyperphosphorylated 4R tau in tubular or straight filaments, in brain regions different from AD. Tau PET depositions have been found in patients with frontotemporal lobar degeneration such as progressive supranuclear palsy and corticobasal degeneration in the basal ganglia, thalamus, midbrain, and dentate nucleus (Chiotis et al., 2016; Kikuchi et al., 2016; Cho et al., 2017; Ishiki et al., 2017; Smith et al., 2017). A recent study found that ^18^F-AV-1451 deposition could differentiate dementia with Lewy bodies from AD (Kantarci et al., 2017). These studies show the utility of tau PET in evaluation of AD and non-AD pathologies.

However, off-target binding has been continuously reported for first-generation tau PET tracers. Binding affinity to β-sheet structures of ^ 18^F-THK5117 showed increased binding in the subcortical white matter (WM) retention (Harada et al., 2015). Studies have shown off-target bindings of ^18^F-AV-1451 in the choroid plexus due to the identification of the tau tangle-like structures (Ikonomovic et al., 2016; Johnson et al., 2016; Ossenkoppele et al., 2016). In particular, ^18^F-THK5351 has limited utility as a sole biomarker of AD-related tauopathy due to its binding to monoamine oxidase-B (MAO-B; Ng et al., 2017; Harada et al., 2018). A recent study has undertaken a cluster analysis based on the regional uptake of ^18^F-AV-1451 (Whitwell et al., 2018). Although a cautious interpretation is needed in tau PET studies due to these non-specific binding properties, tau PET provides valuable evidence of tau pathology *in vivo*.

Multidomain biomarker analyses based on neurodegeneration, tau, and amyloid together for AD subtypes may provide further insights into the subordinate characteristics of AD. AD subtypes have been previously defined in studies using cortical atrophy in MRI (Shiino et al., 2006; Noh et al., 2014; Whitwell et al., 2018), postmortem neurofibrillary tangle counts (Murray et al., 2011), and tau retention in PET scans (Whitwell et al., 2018), but it has not been evaluated with *in vivo* multimodal imaging scans. We sought to investigate whether AD dementia can be categorized into subgroups using the multimodal method comprising 3T MRI, tau PET, and amyloid PET, and whether clinical characteristics are associated with each subtype.

## Materials and Methods

### Participants

A total of 191 participants who had been clinically diagnosed with AD dementia or normal controls (NC) were prospectively recruited from March 2015 to November 2017. All participants underwent 3.0-Tesla MRI, ^18^F-THK5351 PET scans, and ^18^F-Flutemetamol (FLUTE) PET scans and completed neuropsychological tests at the Memory Clinic at Gachon University Gil Medical Center. Of the 191 participants, 37 participants with Clinical Dementia Rating (CDR) >1 were excluded from the study to avoid the effects of disease progression. Thus, 154 participants including patients with AD dementia with CDR ≤1 (*n* = 88) and NC (*n* = 66) were included in this study. Please note that total of 143 participants (AD dementia = 83 and NC = 60) were used since 11 participants were excluded in the quality control step as described in the “Materials and Methods” section.

AD dementia patients were recruited from memory disorder clinic at Samsung Medical Center or Gachon University Gil Medical Center and had been diagnosed with probable AD according to the National Institute of Neurological and Communicative Disorders and Stroke and the AD and Related Disorders Association (McKhann et al., 1984). Diagnoses were confirmed by follow-up for more than 1 year by a neurologist with more than 30 years of experience (DN) and a neurologist with more than 10 years clinical and research experience (YN). The AD patients were classified into early-onset AD (onset age <65) and late-onset AD (onset age ≥65). Patients were excluded if they had structural abnormalities in MRI such as intracranial hemorrhage, cerebral, cerebellar, or brainstem infarction, traumatic brain injury, hydrocephalus, tumors, severe WM hyperintensity, WM hyperintensity associated with radiation, multiple sclerosis, or vasculitis. Other causes of dementia were ruled out with laboratory tests such as complete blood count, folate levels, vitamin B12, thyroid function, metabolic profile, and syphilis serology. Patients with familial AD and vascular dementia were not included in the study. APOE4 genotyping for all participants was obtained.

The 66 participants in the NC group were either spouse of the patients or healthy volunteers from the community who did not have subjective memory complaints and objective cognitive decline. All of them had a CDR score of 0 and normal results on neuropsychological tests (defined as within 1.5 standard deviations of age- and education-corrected normative mean). Participants were excluded if they had structural abnormalities in MRI such as intracranial hemorrhage, cerebral, cerebellar, or brainstem infarction, traumatic brain injury, hydrocephalus, tumors, severe WM hyperintensity, WM hyperintensity associated with radiation, multiple sclerosis, or vasculitis.

Written informed consent was obtained from all participants and the study was approved by the Institutional Review Board of Gachon University Gil Medical Center.

### Neuropsychological Assessment

Mini-Mental State Examination (MMSE), CDR, CDR-sum of boxes (CDR-SOB) results were obtained and detailed neuropsychological function tests including attention, praxis, frontal/executive function, visual and verbal memory, language, visuoconstructive ability, and elements of Gerstmann syndrome were evaluated in all participants. Detailed items of the comprehensive test battery (Kang and Na, 2003) have been described in our previous study (Lee et al., 2018).

### Image Acquisition

All participants underwent brain MRI using a 3.0-T MRI scanner (Verio, Siemens with a Siemens matrix coil) as described in our previous study (Kang et al., 2017). Both ^18^F-THK5351 and ^18^F-FLUTE PET scans were acquired using a Siemens Biograph 6 Truepoint PET/computed tomography scanner (Siemens, Knoxville, TN, USA) with a list-mode emission acquisition. THK5351 scans were acquired for 20 min starting from 50 min after the injection of 185MBq of ^18^F-THK5351 intravenously (50–0 min), which was synthesized and radiolabeled in Gachon University Neuroscience Research Institute. ^18^F-FLUTE emission scans were acquired for 20 min starting from 90 min after the intravenous injection of 185 MBq of 18F-FLUTE (90–110 min), purchased from Carecamp Inc. The mean interval between the PET scans was 13.94 ± 14.02 days and detailed data of interval between the two PET scans are presented in Appendix 1 in Supplementary Materials. Attenuation correction was performed with a low-dose CT scan and data reconstruction was performed with a 2D ordered subset expectation maximization algorithm (eight iterations and 16 subsets).

### Image Processing

#### Cortical Surface Reconstruction and Cortical Thickness Measurement

We followed the CIVET pipeline^1^ (version 2.1). Briefly, each subject’s T1-weighted image was corrected for intensity inhomogeneity and linearly registered to the Montreal Neurological Institute-152 template to bring the images into a common space (Collins et al., 1994). The images were then tissue classified into WM, gray matter (GM), or cerebrospinal fluid (CSF; Zijdenbos et al., 2002) and the inner (WM/GM boundary) and the outer (GM/CSF boundary) cortical surfaces were extracted resulting in 40,962 vertex points per hemisphere (Kim et al., 2005). To obtain vertex-correspondence between individuals, surfaces were registered to an unbiased group template by matching the sulcal folding pattern (Lyttelton et al., 2007). The registered surfaces were transformed back into each patient’s native space, and cortical thicknesses were calculated as the Euclidean distance between the linked vertices of the inner and outer surfaces (Lerch et al., 2005). The measured cortical thickness was smoothed using a 30 mm full width at half maximum (FWHM) surface-based diffusion smoothing kernel (Chung et al., 2003).

#### Surface-Based Measurement for ^18^F-THK5351 and ^18^F-FLUTE

We rigidly co-registered the PET scans to native T1-weighted images using mutual information as a cost function. The cortical surfaces and tissue classes were linearly registered into the PET scans by applying inverse transform matrices. We performed partial volume correction (PVC) within gray and WM regions using iterative deconvolution with a surface-based anatomically constrained filtering (idSURF) method that uses the representation of the volume between the inner and outer surfaces as a spatial constraint to the PET signal (Funck et al., 2014). The PVC images were normalized to the reference regions resulting in a standardized uptake value ratio (SUVR). We used cerebellum GM (Okamura et al., 2014; Lockhart et al., 2016) and pons (Thurfjell et al., 2014) as low receptor density reference regions in the THK5351 and FLUTE analyses, respectively. The SUVR signal intensities were sampled at 50% of the distance from the inner to the outer surface to minimize partial volume contamination. The measured signals were spatially blurred using a surface-based diffusion smoothing kernel (20 mm FWHM).

#### Image Quality Control

All raw images and the results produced from the pipeline were carefully verified (by two investigators blinded to participant information). We excluded five AD and six NC participants due to MRI motion artifacts and image processing errors in brain masking, tissue classification, and cortical surface extraction.

#### Cluster Analysis

**Figure 1:**
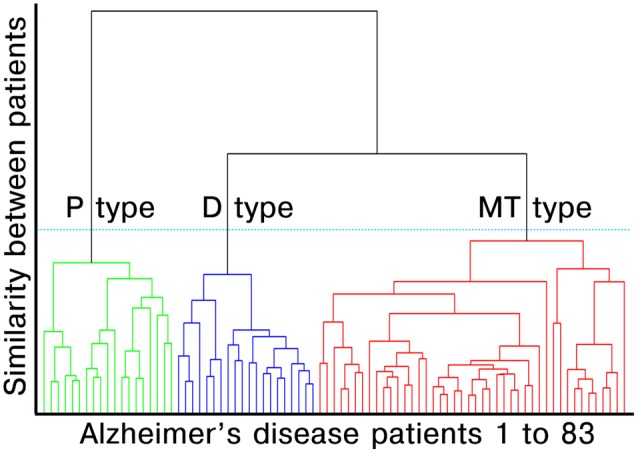
Dendrogram created by surface-based multimodal cluster analysis. AD, Alzheimer’s disease; MT, medial temporal-dominant; P, Parietal-dominant; D, Diffuse atrophy. The distance along the y-axis represents the measure of similarity between patients, such that the shorter the distance, the greater the similarity between patients. The green, blue and red lines represent the clustered subtypes of AD dementia. The three clusters were selected based on Gap statistics (see Appendix 2 in Supplementary Materials).

We performed agglomerative hierarchical clustering analysis using z-scored multimodal imaging features without noncortical regions on the surface model (three features with 79,950 vertices each). The hierarchical clustering method combines pairs of clusters at each step while minimizing the sum of squared errors from the cluster mean (Ward, 1963). Each of the 83 patients with AD dementia was placed in their own cluster and then progressively clustered with others. The AD patients belonging to the same cluster had similar profiles, while those in the different cluster had different profiles. The dendrogram created by the surface-based multimodal cluster analysis is presented in Figure 1. To estimate the optimal cluster number, we used the Gap statistics package available in the R software (version 3.5.1, R Development Core Team). Gap compares changes in the total intra-cluster variation for the different number of clusters with the expected values under the null reference distribution of the data (i.e., a distribution with no obvious clustering; Tibshirani et al., 2001). The optimal cluster number was three which yielded the maximum Gap statistic (Appendix 2 in Supplementary Materials). The number of Monte Carlo bootstrap iterations for the computation was set to 2,000.

#### Regions of Interest-Based Measurement

Volume-weighted SUVR values of images were averaged for regions of interest (ROIs) predefined using the Desikan-Killiany-Tourville atlas (Desikan et al., 2006). The value of the FLUTE cortical composite ROI was calculated with cortical SUVRs including the frontal, parietal, lateral temporal, and anterior and posterior cingulate cortices (Thurfjell et al., 2014) and that of the THK5351 global ROI was based on AD-related regions. Detailed regions are presented in Appendix 3 in Supplementary Materials.

### Statistical Analysis

To estimate topographical abnormalities in AD dementia subtypes, we applied general linear models and random field theory using the SurfStat toolbox (Worsley et al., 2009). The three subtypes were compared to the NC group using a general linear model adjusting for age, sex, and years of education. Intracranial volume was included in the cortical thickness analysis. We mapped the effect size (Cohen’s *d*, adjusted for the nuisance variables) within the significant region reaching a *p*-vertex < 0.05 (random field theory) on the standard cortical surface.

Group comparisons of demographic and clinical characteristics between AD and NC were performed using an independent *t*-test for continuous variables and chi-square test for categorical variables. In comparisons among the three AD subtypes, one-way analysis of variance followed by Bonferroni *post hoc* test was used (*p* < 0.05). Mean cortical thickness, THK SUVR, and FLUTE SUVR were compared between AD and NC using independent *t*-test and analysis of covariance (covariance: age, gender, and years of education) followed by pairwise comparisons for adjusted means (Bonferroni, *p* < 0.05). Region-wise multiple comparisons were corrected using the Benjamini-Hochberg false discovery rate method (Benjamini and Hochberg, 1995).

## Results

### Subtypes of Alzheimer’s Disease Based on Multimodal Cluster Analysis

**Figure 2:**
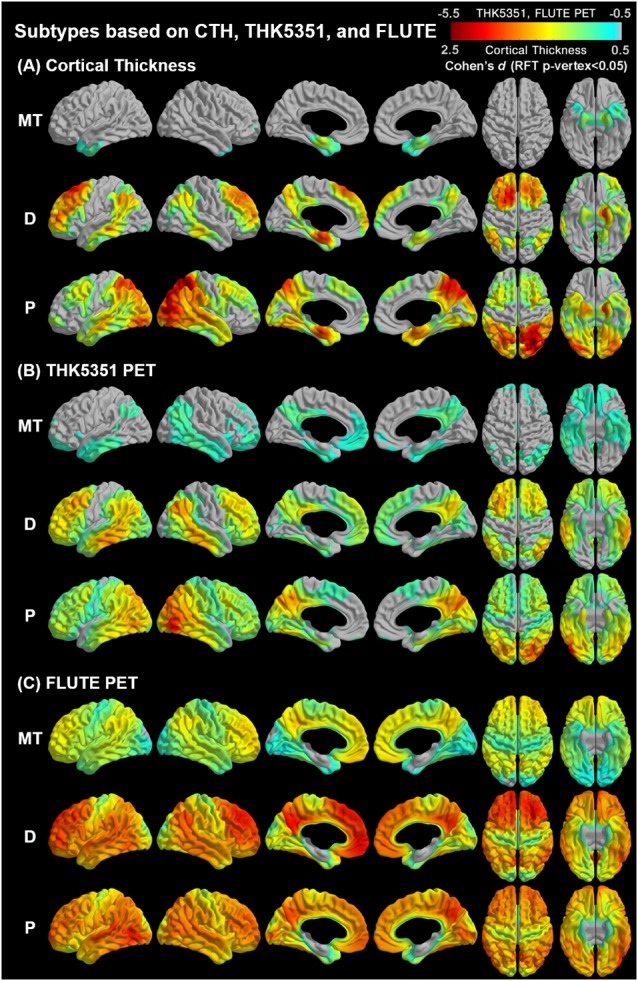
Topographical maps of imaging biomarkers according to AD subtypes. AD, Alzheimer’s disease; MT, Medial temporal-dominant; P, parietal-dominant; D, diffuse atrophy. Comparison of regional **(A)** cortical thickness, **(B)** tau retention, and **(C)** amyloid uptake between identified subtypes and the control group. The color scale indicates the effect size (Cohen’s). Significance was determined based on general linear models controlling for age, sex, and years of education. Intracranial volume was added as a covariate to the cortical thickness analyses. The statistical maps were thresholded using a random field theory (*p*-vertex < 0.05).

Agglomerative hierarchical clustering analysis based on cortical thickness, THK5351 PET, and FLUTE PET yielded three subtypes in the AD patients (Figure 2): medial temporal-dominant subtype (MT, *n* = 44), parietal-dominant subtype (P, *n* = 19), and diffuse atrophy subtype (D, *n* = 20). In the MT subtype, cortical thinning was prominent in the medial temporal cortex. THK retention was most increased in the medial and lateral temporal cortices and slightly in the inferior parietal, precuneus-posterior cingulate (PC-PCC), and frontal cortices. FLUTE retention was identified in some regions of the dorsolateral prefrontal, medial frontal, lateral temporal, parietal cortices, and PC-PCC (Figures 2A–C). In the P subtype, cortical thinning was relatively distinct in the parietal, PC-PCC, and occipital cortices. THK retention was also dominant in the parietal cortex and PC-PCC and FLUTE retention was more pronounced in the parietal, PC-PCC, lateral temporal, frontal, and occipital cortices (Figures 2A–C). In the D subtype, cortical thinning was found in relatively diffuse cortices including the frontal, medial temporal, lateral temporal, inferior parietal, and PC-PCC. THK retention was found in very similar areas where cortical thinning was identified, and amyloid uptake was found in the diffuse brain cortices saving the primary sensorimotor cortex (Figures 2A–C).

The results of ROI-based comparisons of three imaging markers (cortical thickness, THK SUVR, and FLUTE SUVR) among the diagnostic groups are shown in Appendix 4 in Supplementary Materials. Cortical thinning in the superior parietal cortex was most prominent in the P subtype (2.66 ± 0.12 for MT, 2.29 ± 0.24 for P, and 2.56 ± 0.21, *p* < 0.001) but that in the mesial temporal cortex was similar across subtypes (2.80 ± 0.19 for MT, 2.79 ± 0.16 for P, and 2.82 ± 0.18, *p* = 0.831). THK retention in the mesial temporal cortex showed subtle differences across subtypes (2.16 ± 0.28 for MT, 2.15 ± 0.16 for P, and 2.09 ± 0.24, *p* = 0.594) but the P subtype showed dominant THK retention in the superior parietal cortex (1.36 ± 0.13 for MT, 1.98 ± 0.36 for P, and 1.54 ± 0.17, *p* < 0.001). In terms of FLUTE retention, there was no ROI that showed a significant difference between the MT and D subtypes. The P subtype showed greater FLUTE retention than the MT subtype in the inferior parietal areas only (Appendix Table A4-3). The FLUTE retention showed little topographical difference across the three subtypes while the cortical composite SUVR of the P type was significantly greater than that of the MT subtype (Table 1, Appendix Table A4-3).

**Table 1:** Demographic and clinical characteristics of the study population.

Variables				AD subtypes			
	NC (*n* = 60)	AD (*n* = 83)	*p*-value	MT (*n* = 44)	P (*n* = 19)	D (*n* = 20)	*p*-value
Age at scan, years	66.20 ± 11.08	67.55 ± 10.10	0.449	72.34 ± 9.37	59.47 ± 7.21	64.70 ± 8.07	<0.001*^,†,‡^
Onset age	-	64.06 ± 9.81	-	68.75 ± 9.18	56.12 ± 6.09	61.27 ± 8.19	<0.001*^,†,‡^
Sex, female, *n* (%)	29 (48.3%)	59 (71.1%)	0.006*	37 (84.1%)	13 (68.4%)	9 (45%)	0.006*
Education, year	11.85 ± 4.70	8.42 ± 4.53	<0.001*	7.51 ± 4.42	9.18 ± 4.74	9.70 ± 4.35	0.142
Disease duration, month	-	41.98 ± 21.97	-	43.07 ± 23.47	40.26 ± 20.04	41.20 ± 20.78	0.885
Mean CTh, mm	2.47 ± 0.10	2.33 ± 0.10	<0.001*	2.38 ± 0.11	2.25 ± 0.13	2.31 ± 0.14	0.001*^,‡^
Global THK5351 retention	1.31 ± 0.12	1.65 ± 0.19	<0.001*	1.54 ± 0.12	1.81 ± 0.19	1.74 ± 0.18	<0.001*^,†,‡^
Cortical composite FLUTE retention	1.22 ± 0.10	2.13 ± 0.34	<0.001*	2.06 ± 0.26	2.39 ± 0.48	2.24 ± 0.29	0.001*^,‡^
APOE4 carrier, *n* (%)	12 (20.0%)	44 (53.0%)	<0.001*	28 (63.6%)	7 (36.8%)	9 (45.0%)	0.105
MMSE	27.90 ± 2.05	18.73 ± 4.98	<0.001*	19.39 ± 4.71	17.59 ± 4.54	18.21 ± 5.93	0.399
CDR-SOB	-	4.31 ± 1.89	-	3.90 ± 1.88	4.95 ± 1.74	4.60 ± 1.93	0.094

NC, normal control; AD, Alzheimer’s disease; MT, medial temporal-dominant subtype; P, parietal-dominant subtype; D, diffuse atrophy subtype; CTh, cortical thickness; FLUTE, flutemetamol; SUVR, standardized uptake value ratio; MMSE, Mini-Mental State Examination; CDR-SOB, Clinical Dementia Rating-Sum of Boxes. Independent t-test was used for comparison between NC and AD. One-way analysis of variance was used for comparison among AD subtypes followed by a Bonferroni *post hoc* test. Chi square test (χ^2^) was used for nominal variables. Data are shown as mean ± standard deviation or number (%). *Significant *p*-values between groups (*p* < 0.05). ^†^Significant difference (*p* < 0.05) between MT and D subtypes. ^‡^Significant difference (*p* < 0.05) between MT and P subtypes.

Meanwhile, clustering analyses based on cortical thickness and/or THK5351 showed less distinct classification than the present result (Appendix 5 in Supplementary Materials). The analyses with two or four subtypes also showed less significant group differences than the present result (Appendix 6 in Supplementary Materials).

### Demographic and Clinical Characteristics

The demographics and clinical information showed distinct features among each subtype (Table 1). The MT subtype patients were older than the other subtypes (72.34 ± 9.37 for MT, 59.47 ± 7.21 for P, and 64.70 ± 8.07 for D, *p* < 0.001), and the percentage of females was greater [37 (84.1%) for MT, 13 (68.4%) for P, and 9 (45%) for D, *p* = 0.006]. The P subtype patients had the earliest onset age (68.75 ± 9.18 for MT, 56.12 ± 6.09 for P, and 61.27 ± 8.19 for D, *p* < 0.001) and were the youngest among the subtypes. There were no significant differences in MMSE or CDR-SOB among the three AD subtypes (*p* = 0.399 for MMSE and *p* = 0.094 for CDR-SOB; Table 1).

### Neuropsychological Test Performances

The neuropsychological scores were different among each subtype (Table 2). The K-BNT and verbal memory (SVLT delayed recall and recognition) scores showed no significant difference between the subtypes. The P subtype patients showed poorer performance in attention (digit span backward), visuospatial function (RCFT copy), visual memory (RCFT immediate recall, delayed recall, and recognition), and frontal executive function (COWAT animal/supermarket/phonemic total, Stroop test color reading, and TMT-A/B) than the MT type patients. Patients in the D and P subtypes showed a similar decline in frontal executive function but patients in the D subtype showed better performance than those in the P subtype in the RCFT copy test.

**Table 2:** Neuropsychological test results for AD subtypes.

		MT subtype (*n* = 44)	P subtype (*n* = 19)	D subtype (*n* = 20)	*p*-value
Attention
	Digit span forward	0.15 ± 1.19	−1.08 ± 1.48	−0.22 ± 0.92	0.002*^,‡^
	Digit span backward	−0.40 ± 1.07	−1.94 ± 1.41	−1.43 ± 1.23	<0.001*^,†,‡^
Language function
	K-BNT	−1.53 ± 1.44	−2.79 ± 3.05	−1.82 ± 2.22	0.113
Visuospatial function
	RCFT copy	−0.24 ± 1.46	−9.01 ± 6.40	−3.72 ± 3.25	<0.001*^,†,‡,§^
Memory
	SVLT, immediate recall	−1.39 ± 0.85	−2.38 ± 1.23	−2.26 ± 1.25	0.001*^,†,‡^
	SVLT, delayed recall	−2.13 ± 0.62	−2.48 ± 0.81	−2.46 ± 1.03	0.154
	SVLT, recognition	−1.75 ± 1.38	−2.50 ± 1.46	−2.55 ± 1.53	0.062
	RCFT, immediate recall	−1.35 ± 0.78	−2.10 ± 0.67	−1.74 ± 0.72	0.002*^,‡^
	RCFT, delayed recall	−1.63 ± 0.87	−2.37 ± 0.71	−1.97 ± 0.96	0.010*^,‡^
	RCFT, recognition	−1.54 ± 1.17	−2.44 ± 1.24	−2.28 ± 1.96	0.039*
Frontal/executive function
	COWAT, animal	−1.52 ± 0.91	−2.25 ± 0.70	−2.27 ± 1.00	0.002*^,†,‡^
	COWAT, supermarket	−1.09 ± 0.91	−1.93 ± 0.82	−1.97 ± 0.76	<0.001*^,†,‡^
	COWAT, phonemic total	−0.56 ± 1.00	−1.71 ± 1.27	−1.67 ± 0.98	<0.001*^,†,‡^
	Stroop test, color reading	−1.09 ± 0.93	−2.90 ± 1.33	−2.50 ± 1.13	<0.001*^,†,‡^
	TMT-A	−0.93 ± 2.01	−11.07 ± 12.60	−6.13 ± 9.54	<0.001*^,†,‡^
	TMT-B	−3.70 ± 3.54	−10.09 ± 7.92	−6.63 ± 4.69	<0.001*^,‡^

AD, Alzheimer’s disease; MT, Medial temporal-dominant; P, parietal-dominant; D, diffuse atrophy; K-BNT, Korean version of the Boston naming test; RCFT, Rey-Osterrieth complex figure test; SVLT, Seoul verbal learning test; COWAT, controlled oral word association test; TMT-A/B, trail making test type A/B. Data are presented as mean ± standard deviation. All data are z-scores derived on age- and education-adjusted norms. Analysis of variance followed by Bonferroni *post hoc* test was used. *Significant *p*-values (*p* < 0.05). ^†^Significant difference (*p* < 0.05) between MT and D subtypes. ^‡^Significant difference (*p* < 0.05) between MT and P subtypes. ^§^Significant difference (*p* < 0.05) between P and D subtypes.

## Discussion

In this study, we performed agglomerative hierarchical cluster analysis based on cortical thickness using 3T MRI, THK5351 PET, and FLUTE PET images in a group of patients with early stages of AD dementia and identified three subtypes: MT, P, and D subtypes. We also found heterogeneous topographical patterns of the three imaging biomarkers, demographic characteristics, and cognitive functions according to the AD subtypes.

Three subtypes in AD dementia were generated incorporating cortical atrophy, tau deposition, and amyloid deposition. The MT (53%), P (23%), and D (24%) subtypes are considered to be analogous to the three pathological subtypes, limbic-predominant (14.3%–21.6%), hippocampal-sparing (10.7%–19.3%), and typical AD (47.4%–70.6%) identified in previous pathologic (Murray et al., 2011; Janocko et al., 2012) and MRI (Whitwell et al., 2012) studies from another study group. Their recent tau PET study also identified three clusters according to AV-1451 uptake in the entorhinal and neocortices; entorhinal low/cortical low, entorhinal low/cortical high, entorhinal high/cortical high (Whitwell et al., 2018) and they largely corresponded to MT, P, and D subtypes in our study, respectively. The lower frequency of the D subtype (24%) in our study compared to the previous studies (47.4%–70.6%) may be attributable to the participant characteristics. There was a lower proportion of late-onset AD patients in our study participants compared to the general AD population, and the methods we used in this study are dependent on the study population.

Of the three subtypes, the P subtype was characterized as having a younger age and a lower tendency for APOE4 frequency compared to the MT and D subtypes (Table 1). These characteristics were similar to those of early-onset AD (Licht et al., 2007). The P subtype is in line with the hippocampal-sparing subtype in previous studies with features of younger age, atypical clinical presentation, and lower APOE4 frequency (Murray et al., 2011). In contrast, the MT subtype was characterized by older age, a higher percent of females, and a higher tendency for APOE4 frequency (Table 1). These results are similar with those found in previous studies showing higher alterations in the hippocampus of the female brain due to age-related estrogen reduction (Fester et al., 2012) and region-specific effects of APOE4 in the medial temporal lobe (Hashimoto et al., 2001).

A novel finding in our study is that the topographical heterogeneity was manifested based on the multimodal imaging biomarkers MRI, tau PET, and amyloid PET (Figure 2). Cortical atrophy and THK retention showed similar topography at each subtype. As for the MT subtype, cortical atrophy was observed in the medial temporal cortex and THK retention was also observed mainly in the medial temporal and basal forebrain regions which represent the earliest pathologic changes (Braak and Braak, 1991). In the P subtype, both cortical atrophy and tau retention were observed in the temporoparietal regions related to the subsequent state of AD (Braak and Braak, 1991). In the D subtype, cortical atrophy and tau retention were observed at comparable extent both in the medial temporal region and the diffuse neocortical regions. This parallel topography between tau PET and MRI scans according to AD subtypes is supported by earlier and recent studies. Autopsy studies have documented that cortical atrophy proceeds similarly to neurofibrillary tangle pathology (Braak and Braak, 1991; Whitwell et al., 2008) and *in vivo* imaging studies have also reported similar distribution between cortical atrophy and tau retention in patients with typical and atypical AD (Xia et al., 2017; Nasrallah et al., 2018; Whitwell et al., 2018). A previous study also suggested that CSF tau could not correspond to the cortical atrophic patterns because CSF results show only pooled information on tau in the whole brain (Hwang et al., 2015). Thus, our result may suggest that neurofibrillary tangle formation and cortical thinning can have distinct topographic patterns within AD and that MRI and tau PET scans show this considerably. On the other hand, amyloid PET findings showed only a higher uptake in the P subtype without distinct topographic patterns differentiating AD subtypes. This can be supported by the phases of Aβ deposition which are different from the cortical thickness (Chételat et al., 2010) and tau retention (Thal et al., 2002) in patients with AD. Aβ deposition is known to precede neurodegeneration and clinical decline (Jack et al., 2009) and does not correlate with cortical atrophy in AD, unlike tau retention (Josephs et al., 2008). Cortical composite SUVR for the P type was significantly greater than that of the MT subtype. This might be related to the characteristics of the subgroup population. The mean age of the MT subtype was older than the P subtype, and the main portion of patients in the MT subtype was late-onset AD. Among the late-onset AD patients, there have been known mixed pathologies in the brain such as TDP-43, argyrophilic grain disease (Ferrer et al., 2008; Landau et al., 2016), aging-related tau astrogliopathy, or hippocampal sclerosis (Nelson et al., 2011; Cairns et al., 2015).

Cognitive function differed according to the AD subtypes (Table 2). In the P subtype, visuoconstruction was significantly impaired compared to the MT and D subtypes and it is known that visuospatial cognition is predominantly mediated by parietal lobe function (Possin, 2010). Poorer results for attention, visual memory, and frontal executive function were also found in the P subtype compared to the MT subtype. The worst cognitive function and non-amnestic features in the P subtype may be characteristics of early-onset AD. However, the three subtypes did not differ in the language (K-BNT) and verbal memory tests (SVLT delayed recall and recognition).

We note several limitations to our study. We could not undertake pathologic confirmation because we only used imaging biomarkers. The cross-sectional design of the study without longitudinal follow-up is another limitation in that progression of each subtype is unknown. As the study participants were younger and had a greater proportion of early-onset AD compared to the general population, the distribution and characteristics of the AD subtypes could be affected. As noted, the limitation of the THK5351 tracer itself should be regarded in our study because THK5351 PET shows binding to MAO-B (Ng et al., 2017; Harada et al., 2018). THK5351 is known to trace not only neurofibrillary tangles but a combination of neurofibrillary tangles and reactive astrocytes. Although the analysis in this study did not include the subcortical structure that is most influenced by MAO-B availability, it should be carefully interpreted due to MAO-B distribution throughout the whole brain (Ng et al., 2017; Harada et al., 2018).

## Conclusion

Our surface-based multimodal imaging cluster analysis framework has revealed three distinct subtypes among AD patients in terms of the distribution of cortical atrophy, THK5351 retention, and FLUTE retention. We used three crucial imaging biomarkers and identified the three subtypes of AD consistent with the previous pathologic or imaging studies and suggest that multimodal *in vivo* imaging biomarkers may differentiate the subtypes of AD, mainly by the tau deposition and cortical atrophic pattern. Future work will focus on the combinations of various biomarkers more specific to AD pathology and provide further evidence of the multifaceted basis of AD. Consideration for topographic heterogeneity may be important when planning future preventative and treatment strategies because the AD subtypes may have different courses of disease progression and different responses to treatment. In addition, since the cluster analysis algorithm is dependent on the characteristics of the participants, further evaluation in a large cohort is needed.

## Data Availability

The data set generated and/or analyzed during the current study are available from the corresponding author, Prof. Young Noh on reasonable request.

## Ethics Statement

Written informed consent was obtained from all participants and the study was approved by the Institutional Review Board of Gachon University Gil Medical Center.

## Author Contributions

YN, DN, and AE conceptualized and designed the study. SJ and JK drafted the manuscript. JK, KP, Y-BL, BY, DN, and YN acquired the data. SJ, JK, SS, HJ, TF, AE, and YN analyzed the data. S-YL, TI, and NO also contributed to the PET data acquisition and analyses. YN revised the manuscript for intellectual content. All authors reviewed and approved for publication.

## Conflict of Interest Statement

The authors declare that the research was conducted in the absence of any commercial or financial relationships that could be construed as a potential conflict of interest.

## Footnotes

^1^http://mcin.ca/civet
