# Supplementary material for: Topographical Heterogeneity of Alzheimer’s Disease Based on MR Imaging, Tau PET, and Amyloid PET
Source: Front Aging Neurosci. 2019 Aug 20;11:211. doi: 10.3389/fnagi.2019.00211 (PMC6710378; doi:10.3389/fnagi.2019.00211)
Supplement: Supplementary file 1 [file Data_Sheet_1.doc]

**Supplementary Materials**

**Appendix 1. Interval between FLUTE and THK PET scans**


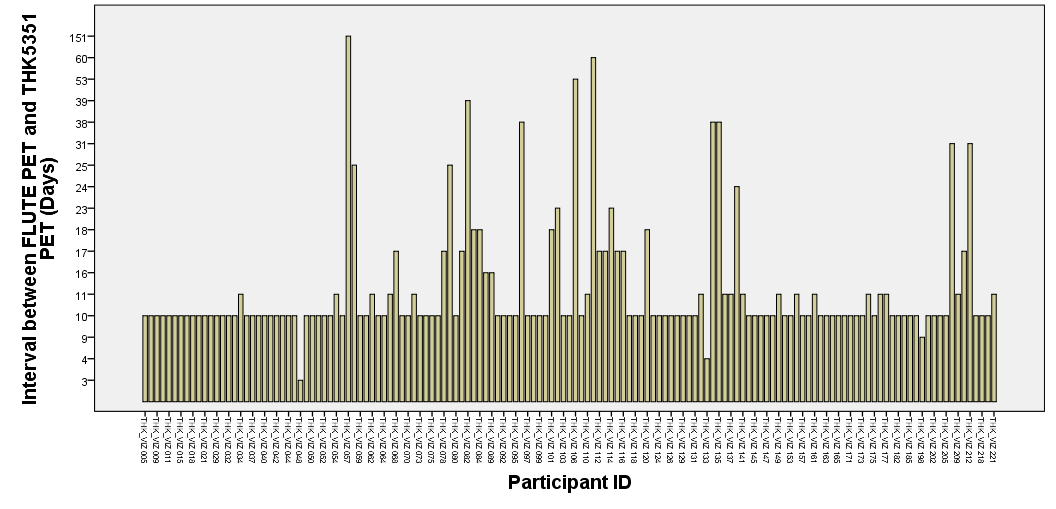


The interval between two PET scans ranges 3 to 151 days. The mean interval is 13.94 ± 14.02 days.

**Appendix 2. The determination of the optimal cluster number.**


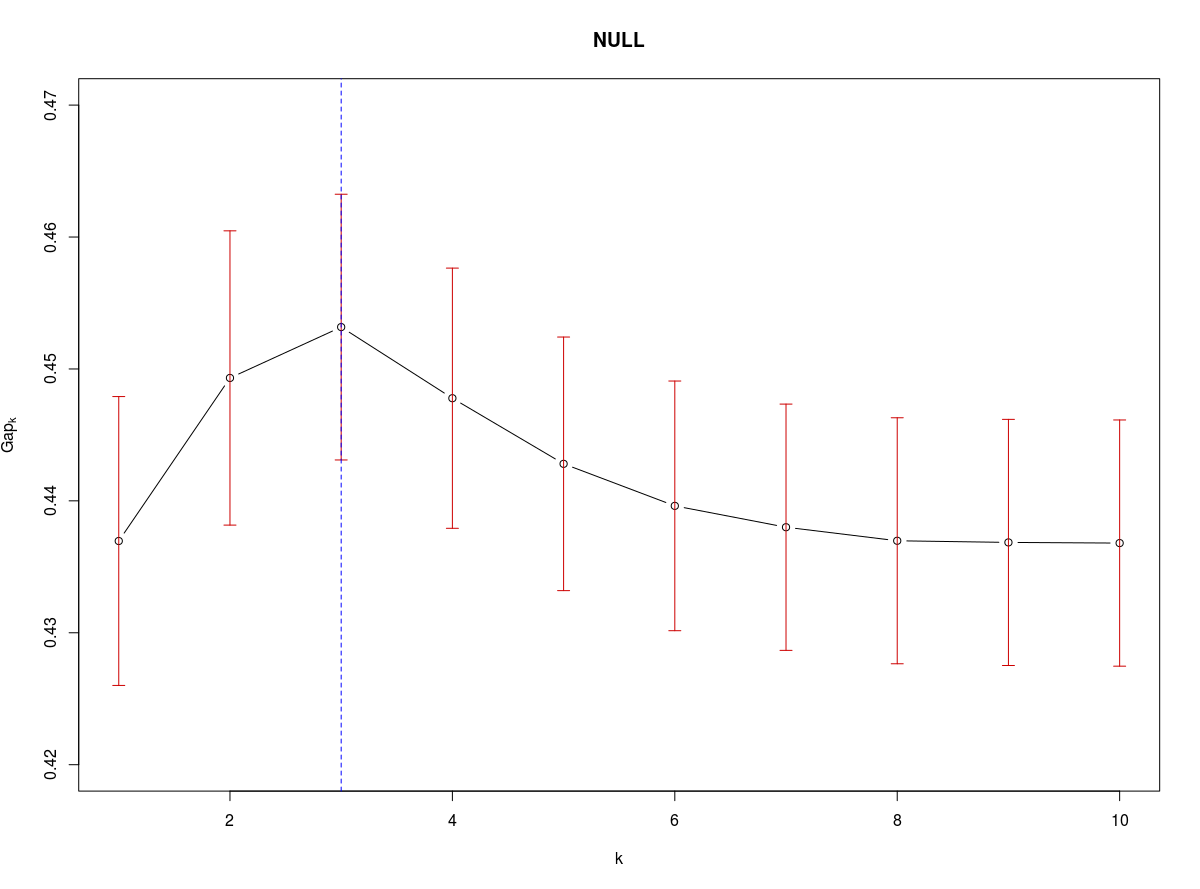


The optimal cluster number was three which yielded the maximum Gap statistic (k = 3, blue dotted line). The number of Monte Carlo bootstrap iterations for the computation of the Gap statistics was set to 2,000. The red line indicates standard error.

**Appendix 3. Region of interest-based measurement**

Twenty-two ROIs were used: The prefrontal, orbitofrontal, sensorimotor, anterior cingulate, superior parietal, inferior parietal, precuneus, posterior cingulate, occipital, superior temporal, middle temporal, inferior temporal, mesial temporal, entorhinal, parahippocampus, fusiform gyrus, lingual gyrus, insula, transverse temporal, lateral temporal, composite cortical, and global data.

The value of the THK5351 global ROI was calculated based on AD-related regions including the lateral frontal orbitalis, lateral frontal triangularis, lateral frontal opercularis, rostral middle frontal, superior frontal gyrus, caudal middle frontal, medial orbitofrontal, superior parietal, inferior parietal, supramarginal gyrus, precuneus, cuneus, pericalcarine, inferior occipital cortex, inferior temporal, middle temporal, superior temporal, parahippocampal, entorhinal cortex, caudal anterior cingulate, rostral anterior cingulate and posterior cingulate.

**Appendix 4. ROI-based comparison of three imaging markers among the diagnostic groups**

| **Table A4-1**. Comparisons of regional cortical thickness | | | | | | | | | | | |
| --- | --- | --- | --- | --- | --- | --- | --- | --- | --- | --- | --- |
| **ROIs** | **NC**  **(*n* = 60)** | **AD**  **(*n* = 83)** | ***p* value** |  | **AD subtypes** | | | | **Pairwise comparison between AD subtypes, *p* value** | | |
|  |  |  |  |  | **MT (*n* = 44)** | **P (*n* = 19)** | **D (*n* = 20)** | ***p* value** | **MT vs D** | **MT vs P** | **P vs D** |
| Prefrontal | 2.97 ± 0.07 | 2.84 ± 0.12 | <0.001 ^a^ |  | 2.88 ± 0.10 | 2.81 ± 0.13 | 2.79 ± 0.13 | 0.006 ^a^ | 0.010 | 0.108 | >0.999 |
| Orbitofrontal | 2.84 ± 0.09 | 2.73 ± 0.09 | <0.001 ^a^ |  | 2.72 ± 0.08 | 2.73 ± 0.08 | 2.74 ± 0.12 | 0.858 | >0.999 | >0.999 | >0.999 |
| Sensorimotor | 2.79 ± 0.08 | 2.71 ± 0.12 | <0.001 ^a^ |  | 2.73 ± 0.10 | 2.66 ± 0.15 | 2.73 ± 0.12 | 0.085 | >0.999 | 0.102 | 0.214 |
| Anterior cingulate | 2.75 ± 0.09 | 2.70 ± 0.09 | 0.001 ^a^ |  | 2.70 ± 0.09 | 2.68 ± 0.09 | 2.70 ± 0.10 | 0.782 | >0.999 | >0.999 | >0.999 |
| Superior parietal | 2.77 ± 0.11 | 2.55 ± 0.23 | <0.001 ^a^ |  | 2.66 ± 0.12 | 2.29 ± 0.24 | 2.56 ± 0.21 | <0.001 ^a^ | 0.083 | <0.001 ^a^ | <0.001 ^a^ |
| Inferior parietal | 2.89 ± 0.08 | 2.72 ± 0.18 | <0.001 ^a^ |  | 2.81 ± 0.10 | 2.56 ± 0.18 | 2.68 ± 0.20 | <0.001 ^a^ | 0.003 | <0.001 ^a^ | 0.062 |
| Precuneus | 2.90 ± 0.09 | 2.72 ± 0.15 | <0.001 ^a^ |  | 2.78 ± 0.10 | 2.59 ± 0.15 | 2.71 ± 0.16 | <0.001 ^a^ | 0.124 | <0.001 ^a^ | 0.012 |
| Posterior cingulate | 2.82 ± 0.08 | 2.72 ± 0.15 | <0.001 ^a^ |  | 2.75 ± 0.08 | 2.68 ± 0.08 | 2.71 ± 0.13 | 0.047 | 0.503 | 0.052 | >0.999 |
| Occipital | 2.76 ± 0.15 | 2.58 ± 0.18 | <0.001 ^a^ |  | 2.62 ± 0.15 | 2.42 ± 0.19 | 2.62 ± 0.13 | <0.001 ^a^ | >0.999 | <0.001 ^a^ | 0.001 ^a^ |
| Superior temporal | 3.17 ± 0.09 | 3.00 ± 0.16 | <0.001 ^a^ |  | 3.02 ± 0.15 | 2.92 ± 0.17 | 3.01 ± 0.16 | 0.057 | >0.999 | 0.059 | 0.215 |
| Middle temporal | 3.11 ± 0.07 | 2.94 ± 0.16 | <0.001 ^a^ |  | 2.99 ± 0.13 | 2.87 ± 0.16 | 2.91 ± 0.18 | 0.006 ^a^ | 0.109 | 0.010 ^a^ | >0.999 |
| Inferior temporal | 3.16 ± 0.10 | 2.96 ± 0.17 | <0.001 ^a^ |  | 2.99 ± 0.15 | 2.89 ± 0.20 | 2.95 ± 0.17 | 0.150 | >0.999 | 0.158 | 0.880 |
| Mesial temporal | 3.07 ± 0.11 | 2.80 ± 0.18 | <0.001 ^a^ |  | 2.80 ± 0.19 | 2.79 ± 0.16 | 2.82 ± 0.18 | 0.831 | >0.999 | >0.999 | >0.999 |
| Entorhinal | 3.52 ± 0.17 | 3.10 ± 0.29 | <0.001 ^a^ |  | 3.08 ± 0.31 | 3.09 ± 0.25 | 3.16 ± 0.29 | 0.595 | 0.944 | >0.999 | >0.999 |
| Parahippocampus | 2.85 ± 0.10 | 2.66 ± 0.13 | <0.001 ^a^ |  | 2.66 ± 0.14 | 2.64 ± 0.12 | 2.66 ± 0.14 | 0.870 | >0.999 | >0.999 | >0.999 |
| Fusiform gyrus | 3.10 ± 0.10 | 2.94 ± 0.15 | <0.001 ^a^ |  | 2.98 ± 0.13 | 2.84 ± 0.18 | 2.96 ± 0.12 | 0.003 ^a^ | >0.999 | 0.002 ^a^ | 0.052 |
| Lingual gyrus | 2.76 ± 0.17 | 2.59 ± 0.15 | <0.001 ^a^ |  | 2.62 ± 0.15 | 2.49 ± 0.16 | 2.61 ± 0.12 | 0.008 ^a^ | >0.999 | 0.008 ^a^ | 0.043 |
| Insula | 3.37 ± 0.11 | 3.23 ± 0.13 | <0.001 ^a^ |  | 3.24 ± 0.12 | 3.20 ± 0.11 | 3.25 ± 0.16 | 0.430 | >0.999 | 0.805 | 0.687 |
| Transverse temporal | 3.00 ± 0.12 | 2.87 ± 0.14 | <0.001 ^a^ |  | 2.89 ± 0.13 | 2.81 ± 0.15 | 2.90 ± 0.14 | 0.058 | >0.999 | 0.105 | 0.094 |
| Lateral temporal | 3.15 ± 0.08 | 2.97 ± 0.16 | <0.001 ^a^ |  | 3.00 ± 0.14 | 2.90 ± 0.17 | 2.96 ± 0.16 | 0.040 | 0.912 | 0.036 | 0.559 |
| ROI = region of interest; NC = normal control; AD = Alzheimer’s disease; MT = medial temporal-dominant; P = parietal-dominant; D = diffuse atrophy  Independent t-test was used for comparison between NC and AD.  Analysis of variance was used for comparison among AD subtypes followed by post hoc test (Bonferroni, *p* < 0.05).  Data are shown as mean ± SD.  ^a^ Significant after Benjamini-Hochberg FDR correction for multiple comparisons (*p* < 0.05). | | | | | | | | | | | |

| **Table A4-2**. Comparisons of regional SUVR of [^18^F]THK PET | | | | | | | | | | | |
| --- | --- | --- | --- | --- | --- | --- | --- | --- | --- | --- | --- |
| **ROIs** | **NC**  **(*n* = 60)** | **AD**  **(*n* = 83)** | ***p* value** |  | **AD subtypes** | | | | **Pairwise comparison between AD subtypes, *p* value** | | |
|  |  |  |  |  | **MT (*n* = 44)** | **P (*n* = 19)** | **D (*n* = 20)** | ***p* value** | **MT vs D** | **MT vs P** | **P vs D** |
| Prefrontal | 1.27 ± 0.12 | 1.56 ± 0.19 | <0.001 ^a^ |  | 1.47 ± 0.12 | 1.59 ± 0.19 | 1.72 ± 0.21 | <0.001 ^a^ | <0.001 ^a^ | 0.030 ^a^ | 0.044 |
| Orbitofrontal | 1.67 ± 0.17 | 1.96 ± 0.20 | <0.001 ^a^ |  | 1.94 ± 0.19 | 1.92 ± 0.16 | 2.05 ± 0.26 | 0.093 | 0.144 | >0.999 | 0.177 |
| Sensorimotor | 1.11 ± 0.10 | 1.25 ± 0.13 | <0.001 ^a^ |  | 1.20 ± 0.10 | 1.34 ± 0.18 | 1.27 ± 0.11 | 0.001 ^a^ | 0.175 | <0.001 ^a^ | 0.229 |
| Anterior cingulate | 1.64 ± 0.18 | 1.84 ± 0.17 | 0.001 ^a^ |  | 1.85 ± 0.17 | 1.78 ± 0.17 | 1.90 ± 0.19 | 0.132 | 0.948 | 0.518 | 0.137 |
| Superior parietal | 1.14 ± 0.09 | 1.54 ± 0.32 | <0.001 ^a^ |  | 1.36 ± 0.13 | 1.98 ± 0.36 | 1.54 ± 0.17 | <0.001 ^a^ | 0.009 ^a^ | <0.001 ^a^ | <0.001 ^a^ |
| Inferior parietal | 1.20 ± 0.10 | 1.62 ± 0.29 | <0.001 ^a^ |  | 1.44 ± 0.13 | 1.95 ± 0.30 | 1.72 ± 0.21 | <0.001 ^a^ | <0.001 ^a^ | <0.001 ^a^ | 0.002 ^a^ |
| Precuneus | 1.30 ± 0.11 | 1.70 ± 0.25 | <0.001 ^a^ |  | 1.56 ± 0.14 | 1.93 ± 0.22 | 1.80 ± 0.26 | <0.001 ^a^ | <0.001 ^a^ | <0.001 ^a^ | 0.147 |
| Posterior cingulate | 1.34 ± 0.13 | 1.63 ± 0.18 | <0.001 ^a^ |  | 1.56 ± 0.13 | 1.70 ± 0.19 | 1.71 ± 0.22 | 0.002 ^a^ | 0.007 ^a^ | 0.013 ^a^ | >0.999 |
| Occipital | 1.19 ± 0.09 | 1.48 ± 0.23 | <0.001 ^a^ |  | 1.36 ± 0.14 | 1.77 ± 0.23 | 1.47 ± 0.16 | <0.001 ^a^ | 0.057 | <0.001 ^a^ | <0.001 ^a^ |
| Superior temporal | 1.44 ± 0.15 | 1.73 ± 0.21 | <0.001 ^a^ |  | 1.66 ± 0.16 | 1.84 ± 0.24 | 1.78 ± 0.20 | 0.001 ^a^ | 0.056 | 0.002 ^a^ | 0.953 |
| Middle temporal | 1.41 ± 0.15 | 1.89 ± 0.29 | <0.001 ^a^ |  | 1.74 ± 0.21 | 2.06 ± 0.32 | 2.04 ± 0.26 | <0.001 ^a^ | <0.001 ^a^ | <0.001 ^a^ | >0.999 |
| Inferior temporal | 1.53 ± 0.15 | 2.03 ± 0.31 | <0.001 ^a^ |  | 1.93 ± 0.28 | 2.18 ± 0.32 | 2.11 ± 0.27 | 0.004 ^a^ | 0.064 | 0.006 ^a^ | >0.999 |
| Mesial temporal | 1.78 ± 0.17 | 2.14 ± 0.25 | <0.001 ^a^ |  | 2.16 ± 0.28 | 2.15 ± 0.16 | 2.09 ± 0.24 | 0.594 | 0.946 | >0.999 | >0.999 |
| Entorhinal | 1.80 ± 0.20 | 2.20 ± 0.33 | <0.001 ^a^ |  | 2.24 ± 0.40 | 2.18 ± 0.18 | 2.14 ± 0.27 | 0.487 | 0.777 | >0.999 | >0.999 |
| Parahippocampus | 1.77 ± 0.16 | 2.10 ± 0.22 | <0.001 ^a^ |  | 2.11 ± 0.24 | 2.13 ± 0.15 | 2.06 ± 0.22 | 0.555 | >0.999 | >0.999 | 0.952 |
| Fusiform gyrus | 1.55 ± 0.14 | 1.94 ± 0.28 | <0.001 ^a^ |  | 1.86 ± 0.26 | 2.15 ± 0.29 | 1.93 ± 0.23 | <0.001 ^a^ | 0.923 | <0.001 ^a^ | 0.025 |
| Lingual gyrus | 1.38 ± 0.11 | 1.60 ± 0.19 | <0.001 ^a^ |  | 1.53 ± 0.15 | 1.77 ± 0.21 | 1.59 ± 0.15 | <0.001 ^a^ | 0.607 | <0.001 ^a^ | 0.003 ^a^ |
| Insula | 1.63 ± 0.19 | 1.83 ± 0.18 | <0.001 ^a^ |  | 1.83 ± 0.17 | 1.80 ± 0.18 | 1.85 ± 0.19 | 0.670 | >0.999 | >0.999 | >0.999 |
| Transverse temporal | 1.23 ± 0.12 | 1.35 ± 0.14 | <0.001 ^a^ |  | 1.32 ± 0.12 | 1.40 ± 0.18 | 1.35 ± 0.15 | 0.111 | >0.999 | 0.110 | 0.650 |
| Lateral temporal | 1.45 ± 0.15 | 1.86 ± 0.25 | <0.001 ^a^ |  | 1.76 ± 0.20 | 2.00 ± 0.27 | 1.95 ± 0.22 | <0.001 ^a^ | 0.005 ^a^ | <0.001 ^a^ | >0.999 |
| Global | 1.31 ± 0.12 | 1.65 ± 0.19 | <0.001 ^a^ |  | 1.54 ± 0.12 | 1.81 ± 0.19 | 1.74 ± 0.18 | <0.001 ^a^ | <0.001 ^a^ | <0.001 ^a^ | 0.571 |
| ROI = region of interest; SUVR = standard uptake value ratio; NC = normal control; AD = Alzheimer’s disease; MT = medial temporal-dominant; P = parietal-dominant; D = diffuse atrophy  Independent *t*-test was used for comparison between NC and AD.  Analysis of variance was used for comparison among AD subtypes followed by post hoc test (Bonferroni, *p* < 0.05).  Data are shown as mean ± SD.  ^a^ Significant after Benjamini-Hochberg FDR correction for multiple comparisons (*p* < 0.05). | | | | | | | | | | | |

| **Table A4-3**. Comparisons of regional SUVR of [^18^F]FLUTE PET | | | | | | | | | | | |
| --- | --- | --- | --- | --- | --- | --- | --- | --- | --- | --- | --- |
| **ROIs** | **NC**  **(*n* = 60)** | **AD**  **(*n* = 83)** | ***p* value** |  | **AD subtypes** | | | | **Pairwise comparison between AD subtypes, *p* value** | | |
|  |  |  |  |  | **MT (*n* = 44)** | **P (*n* = 19)** | **D (*n* = 20)** | ***p* value** | **MT vs D** | **MT vs P** | **P vs D** |
| Prefrontal | 1.20 ± 0.11 | 2.24 ± 0.36 | <0.001 ^a^ |  | 2.12 ± 0.26 | 2.42 ± 0.49 | 2.36 ± 0.32 | 0.002 ^a^ | 0.033 | 0.005 ^a^ | >0.999 |
| Orbitofrontal | 1.33 ± 0.14 | 2.36 ± 0.38 | <0.001 ^a^ |  | 2.26 ± 0.26 | 2.52 ± 0.55 | 2.44 ± 0.36 | 0.023 ^a^ | 0.200 | 0.035 ^a^ | >0.999 |
| Sensorimotor | 1.26 ± 0.12 | 1.89 ± 0.29 | <0.001 ^a^ |  | 1.81 ± 0.21 | 2.08 ± 0.40 | 1.88 ± 0.25 | 0.002 ^a^ | 0.974 | 0.001 ^a^ | 0.067 |
| Anterior cingulate | 1.29 ± 0.14 | 2.21 ± 0.35 | <0.001 ^a^ |  | 2.14 ± 0.27 | 2.31 ± 0.50 | 2.25 ± 0.30 | 0.163 | 0.661 | 0.233 | >0.999 |
| Superior parietal | 1.22 ± 0.11 | 2.13 ± 0.36 | <0.001 ^a^ |  | 2.03 ± 0.26 | 2.39 ± 0.48 | 2.12 ± 0.30 | 0.001 ^a^ | >0.999 | 0.001 ^a^ | 0.038 |
| Inferior parietal | 1.20 ± 0.10 | 2.12 ± 0.34 | <0.001 ^a^ |  | 2.03 ± 0.27 | 2.33 ± 0.45 | 2.13 ± 0.27 | 0.003 ^a^ | 0.756 | 0.002 ^a^ | 0.139 |
| Precuneus | 1.21 ± 0.11 | 2.35 ± 0.37 | <0.001 ^a^ |  | 2.25 ± 0.29 | 2.55 ± 0.48 | 2.38 ± 0.35 | 0.011 ^a^ | 0.519 | 0.009 ^a^ | 0.435 |
| Posterior cingulate | 1.23 ± 0.12 | 2.11 ± 0.33 | <0.001 ^a^ |  | 2.04 ± 0.24 | 2.24 ± 0.48 | 2.12 ± 0.32 | 0.107 | >0.999 | 0.110 | 0.879 |
| Occipital | 1.22 ± 0.11 | 1.83 ± 0.36 | <0.001 ^a^ |  | 1.69 ± 0.29 | 2.13 ± 0.39 | 1.87 ± 0.26 | <0.001 ^a^ | 0.107 | <0.001 ^a^ | 0.030 |
| Superior temporal | 1.27 ± 0.11 | 2.14 ± 0.37 | <0.001 ^a^ |  | 2.02 ± 0.28 | 2.39 ± 0.50 | 2.16 ± 0.25 | 0.001 ^a^ | 0.391 | <0.001 ^a^ | 0.096 |
| Middle temporal | 1.23 ± 0.11 | 2.16 ± 0.40 | <0.001 ^a^ |  | 2.03 ± 0.30 | 2.43 ± 0.53 | 2.22 ± 0.30 | <0.001 ^a^ | 0.161 | <0.001 ^a^ | 0.226 |
| Inferior temporal | 1.26 ± 0.12 | 2.09 ± 0.40 | <0.001 ^a^ |  | 1.95 ± 0.31 | 2.37 ± 0.51 | 2.15 ± 0.29 | <0.001 ^a^ | 0.114 | <0.001 ^a^ | 0.171 |
| Mesial temporal | 1.29 ± 0.14 | 1.58 ± 0.24 | <0.001 ^a^ |  | 1.54 ± 0.19 | 1.66 ± 0.35 | 1.59 ± 0.19 | 0.194 | >0.999 | 0.223 | >0.999 |
| Entorhinal | 1.22 ± 0.13 | 1.44 ± 0.24 | <0.001 ^a^ |  | 1.39 ± 0.19 | 1.52 ± 0.34 | 1.46 ± 0.18 | 0.120 | 0.838 | 0.137 | >0.999 |
| Parahippocampus | 1.35 ± 0.16 | 1.67 ± 0.24 | <0.001 ^a^ |  | 1.63 ± 0.19 | 1.74 ± 0.37 | 1.68 ± 0.19 | 0.252 | >0.999 | 0.300 | >0.999 |
| Fusiform gyrus | 1.31 ± 0.13 | 1.98 ± 0.36 | <0.001 ^a^ |  | 1.85 ± 0.30 | 2.23 ± 0.44 | 2.00 ± 0.24 | <0.001 ^a^ | 0.276 | <0.001 ^a^ | 0.095 |
| Lingual gyrus | 1.28 ± 0.13 | 1.79 ± 0.33 | <0.001 ^a^ |  | 1.67 ± 0.29 | 2.04 ± 0.35 | 1.83 ± 0.24 | <0.001 ^a^ | 0.123 | <0.001 ^a^ | 0.082 |
| Insula | 1.31 ± 0.13 | 2.03 ± 0.34 | <0.001 ^a^ |  | 1.94 ± 0.25 | 2.20 ± 0.52 | 2.06 ± 0.25 | 0.016 ^a^ | 0.526 | 0.014 ^a^ | 0.547 |
| Transverse temporal | 1.18 ± 0.12 | 1.91 ± 0.34 | <0.001 ^a^ |  | 1.82 ± 0.28 | 2.14 ± 0.47 | 1.90 ± 0.20 | 0.002 ^a^ | >0.999 | 0.002 ^a^ | 0.067 |
| Lateral temporal | 1.25 ± 0.11 | 2.13 ± 0.38 | <0.001 ^a^ |  | 2.00 ± 0.30 | 2.40 ± 0.51 | 2.18 ± 0.27 | <0.001 ^a^ | 0.202 | <0.001 ^a^ | 0.147 |
| Cortical composite | 1.22 ± 0.10 | 2.18 ± 0.35 | <0.001 ^a^ |  | 2.06 ± 0.26 | 2.39 ± 0.48 | 2.24 ± 0.29 | 0.001 ^a^ | 0.144 | 0.001 ^a^ | 0.432 |
| ROI = region of interest; SUVR = standard uptake value ratio; NC = normal control; AD = Alzheimer’s disease; MT = medial temporal-dominant; P = parietal-dominant; D = diffuse atrophy  Independent t-test was used for comparison between NC and AD.  Analysis of variance was used for comparison among AD subtypes followed by post hoc test (Bonferroni, *p* < 0.05).  Data are shown as mean ± SD.  ^a^ Significant after Benjamini-Hochberg FDR correction for multiple comparisons (*p* < 0.05). | | | | | | | | | | | |

**Appendix 5. Clustering analyses based on MRI and/or THK5351 PET without FLUTE PET**

(1) MRI or THK only

Topographical map


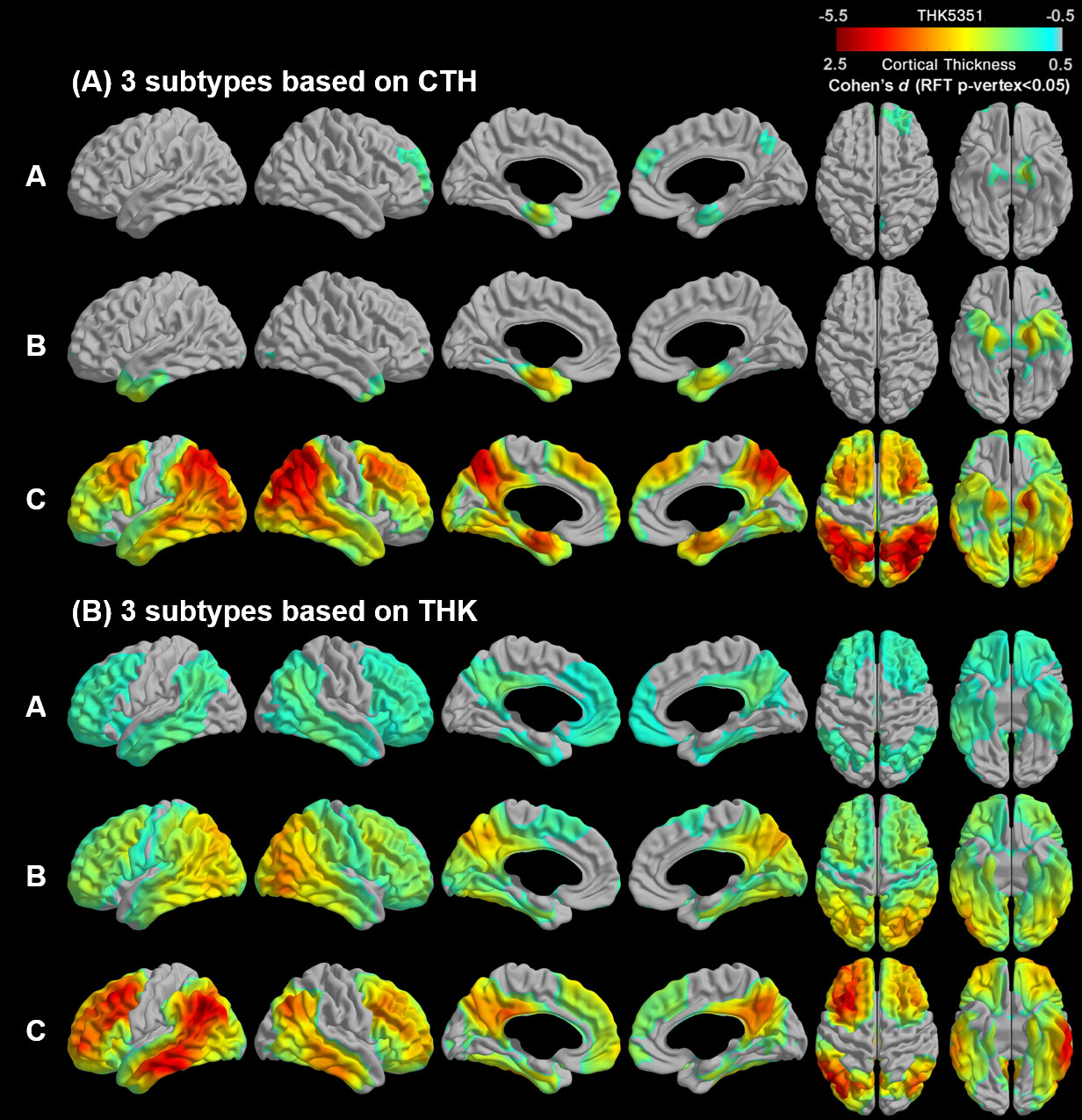


Demographic and clinical data

| **(A) Demographic and clinical characteristics based on MRI only** | | | | |
| --- | --- | --- | --- | --- |
| **Variables** | **AD subtypes** | | | |
|  | **A (*n* = 27)** | **B (*n* = 31)** | **C (*n* = 25)** | ***p* value** |
| Age at scan, years | 66.33 ± 10.22 | 70.87 ± 10.16 | 64.76 ± 9.06 | 0.057 |
| Onset age | 60.75 ± 7.27 | 67.87 ± 10.12 | 62.73 ± 10.32 | 0.016*,† |
| Sex, female, *n* (%) | 21 (77.8%) | 21 (67.7%) | 17 (68.0%) | 0.646 |
| Education, year | 9.00 ± 5.18 | 8.68 ± 4.55 | 7.59 ± 3.88 | 0.500 |
| Disease duration, month | 43.22 ± 14.42 | 35.97 ± 19.24 | 48.08 ± 29.58 | 0.114 |
| Mean CTh, mm | 2.32 ± 0.14 | 2.35 ± 0.12 | 2.33 ± 0.15 | 0.622 |
| Global THK5351 retention | 1.58 ± 0.16 | 1.70 ± 0.20 | 1.66 ± 0.20 | 0.082 |
| Cortical composite FLUTE retention | 2.23 ± 0.43 | 2.13 ± 0.30 | 2.17 ± 0.32 | 0.549 |
| APOE4 carrier, *n* (%) | 14 (51.9%) | 14 (45.2%) | 16 (64.0%) | 0.369 |
| MMSE | 19.15 ± 5.02 | 19.30 ± 5.37 | 17.54 ± 4.41 | 0.383 |
| CDR-SOB | 4.31 ± 2.01 | 4.26 ± 1.88 | 4.36 ± 1.86 | 0.980 |
|  | | | | |
| **(B) Demographic and clinical characteristics based on THK only** | | | | |
| **Variables** | **AD subtypes** | | | |
|  | **A (*n* = 50)** | **B (*n* = 21)** | **C (*n* = 12)** | ***p* value** |
| Age at scan, years | 69.24 ± 10.52 | 62.71 ± 8.63 | 69.00 ± 8.46 | 0.037*,‡ |
| Onset age | 65.98 ± 10.61 | 59.04 ± 7.01 | 64.81 ± 7.81 | 0.022*,‡ |
| Sex, female, *n* (%) | 37 (74.0%) | 17 (81.0%) | 5 (41.7%) | 0.044* |
| Education, year | 7.86 ± 4.43 | 9.52 ± 5.45 | 8.83 ± 2.82 | 0.353 |
| Disease duration, month | 39.08 ± 16.38 | 44.10 ± 26.91 | 50.33 ± 31.05 | 0.249 |
| Mean CTh, mm | 2.33 ± 0.13 | 2.34 ± 0.14 | 2.32 ± 0.13 | 0.919 |
| Global THK5351 retention | 1.63 ± 0.18 | 1.69 ± 0.23 | 1.66 ± 0.18 | 0.544 |
| Cortical composite FLUTE retention | 2.18 ± 0.36 | 2.18 ± 0.35 | 2.18 ± 0.33 | 0.999 |
| APOE4 carrier, *n* (%) | 27 (54.0%) | 10 (47.6%) | 7 (58.3%) | 0.818 |
| MMSE | 19.10 ± 5.11 | 18.05 ± 4.47 | 18.33 ± 5.55 | 0.709 |
| CDR-SOB | 4.24 ± 1.89 | 4.29 ± 1.85 | 4.63 ± 2.11 | 0.821 |
| NC, normal control; AD, Alzheimer’s disease; MT, medial temporal–dominant subtype; P, parietal-dominant subtype; D, diffuse atrophy subtype; CTh, cortical thickness; FLUTE, flutemetamol; SUVR, standardized uptake value ratio; MMSE, Mini-Mental State Examination; CDR-SOB, Clinical Dementia Rating–Sum of Boxes  One-way analysis of variance was used for comparison among AD subtypes followed by a Bonferroni post-hoc test.  Chi square test (χ^2^) was used for nominal variables.  Data are shown as mean ± standard deviation or number (%).  *Significant *p* values between groups (*p* < 0.05).  †Significant difference (*p* < 0.05) between B and C subtypes.  ‡Significant difference (*p* < 0.05) between A and B subtypes. | | | | |

Neuropsychological test results

| **(A) Neuropsychological test results based on MRI only** | | | | |
| --- | --- | --- | --- | --- |
|  | **A (*n* = 27)** | **B (*n* = 31)** | **C (*n* = 25)** | ***p* value** |
| Attention |  |  |  |  |
| Digit Span Forward | -0.14 ± 1.29 | -0.16 ± 1.42 | -0.36 ± 1.24 | 0.812 |
| Digit Span Backward | -0.97 ± 1.23 | -0.80 ± 1.41 | -1.27 ± 1.41 | 0.460 |
| Language and related function |  |  |  |  |
| K-BNT | -1.60 ± 1.52 | -1.96 ± 2.27 | -2.06 ± 2.50 | 0.727 |
| Visuospatial function |  |  |  |  |
| RCFT copy | -2.23 ± 4.86 | -3.05 ± 4.83 | -3.94 ± 5.46 | 0.504 |
| Memory |  |  |  |  |
| SVLT, immediate recall | -1.51 ± 1.14 | -1.81 ± 1.24 | -2.19 ± 0.87 | 0.107 |
| SVLT, delayed recall | -2.22 ± 0.84 | -2.24 ± 0.86 | -2.44 ± 0.61 | 0.553 |
| SVLT, recognition | -2.19 ± 1.60 | -1.99 ± 1.46 | -2.18 ± 1.38 | 0.840 |
| RCFT, immediate recall | -1.56 ± 0.82 | -1.49 ± 0.82 | -1.83 ± 0.72 | 0.289 |
| RCFT, delayed recall | -1.89 ± 0.81 | -1.68 ± 0.96 | -2.14 ± 0.89 | 0.190 |
| RCFT, recognition | -2.05 ± 1.32 | -1.80 ± 1.66 | -1.95 ± 1.35 | 0.814 |
| Frontal executive function |  |  |  |  |
| COWAT, animal | -1.80 ± 0.99 | -1.84 ± 0.88 | -1.97 ± 1.05 | 0.819 |
| COWAT, supermarket | -1.45 ± 0.90 | -1.38 ± 1.07 | -1.70 ± 0.85 | 0.459 |
| COWAT, phonemic total | -0.77 ± 1.32 | -1.30 ± 1.11 | -1.17 ± 1.14 | 0.255 |
| Stroop test, color reading | -1.80 ± 1.28 | -1.63 ± 1.40 | -2.06 ± 1.30 | 0.531 |
| TMT-A | -4.31 ± 9.87 | -5.03 ± 9.33 | -3.54 ± 5.52 | 0.829 |
| TMT-B | -5.36 ± 5.72 | -5.67 ± 5.72 | -6.57 ± 5.52 | 0.755 |
|  | | | | |
| **(B) Neuropsychological test results based on THK only** | | | | |
|  | **A (*n* = 50)** | **B (*n* = 21)** | **C (*n* = 12)** | ***p* value** |
| Attention |  |  |  |  |
| Digit Span Forward | -0.24 ± 1.40 | -0.39 ± 1.14 | -0.22 ± 0.90 | 0.445 |
| Digit Span Backward | -0.89 ± 1.27 | -1.38 ± 1.48 | -0.78 ± 1.46 | 0.345 |
| Language and related function |  |  |  |  |
| K-BNT | -2.00 ± 1.90 | -1.74 ± 2.59 | -1.50 ± 2.27 | 0.743 |
| Visuospatial function |  |  |  |  |
| RCFT copy | -2.53 ± 4.76 | -4.90 ± 6.12 | -1.94 ± 2.91 | 0.165 |
| Memory |  |  |  |  |
| SVLT, immediate recall | -1.73 ± 1.23 | -2.01 ± 0.81 | -1.93 ± 1.19 | 0.626 |
| SVLT, delayed recall | -2.17 ± 0.83 | -2.58 ± 0.75 | -2.30 ± 0.47 | 0.153 |
| SVLT, recognition | -2.11 ± 1.52 | -2.27 ± 1.40 | -1.82 ± 1.40 | 0.729 |
| RCFT, immediate recall | -1.50 ± 0.85 | -1.95 ± 0.77 | -1.49 ± 0.32 | 0.103 |
| RCFT, delayed recall | -1.78 ± 0.92 | -2.13 ± 0.98 | -1.86 ± 0.51 | 0.348 |
| RCFT, recognition | -1.94 ± 1.60 | -1.80 ± 1.30 | -2.03 ± 1.06 | 0.902 |
| Frontal executive function |  |  |  |  |
| COWAT, animal | -1.86 ± 0.94 | -1.86 ± 1.12 | -1.87 ± 0.96 | 0.999 |
| COWAT, supermarket | -1.36 ± 1.01 | -1.70 ± 0.85 | -1.73 ± 0.77 | 0.292 |
| COWAT, phonemic total | -1.06 ± 1.23 | -1.23 ± 1.27 | -0.98 ± 0.96 | 0.825 |
| Stroop test, color reading | -1.76 ± 1.33 | -2.15 ± 1.38 | -1.43 ± 1.24 | 0.351 |
| TMT-A | -4.50 ± 9.16 | -5.43 ± 8.57 | -2.03 ± 5.07 | 0.578 |
| TMT-B | -5.82 ± 5.54 | -7.49 ± 6.31 | -3.19 ± 3.75 | 0.133 |
| AD, Alzheimer’s disease; MT, Medial temporal-dominant; P, parietal-dominant; D, diffuse atrophy; K-BNT, Korean version of the Boston naming test; RCFT, Rey-Osterrieth complex figure test; SVLT, Seoul verbal learning test; COWAT, controlled oral word association test; TMT-A/B, trail making test type A/B  Data are presented as mean ± standard deviation. All data are z-scores derived on age- and education-adjusted norms.  Analysis of variance followed by Bonferroni post hoc test was used. | | | | |

The results of clustering analyses based on MRI or THK alone. In MRI-based analysis, the differences of age, sex, mean cortical thickness, global THK retention, and FLUTE composite retention between subtypes were not significant which were found in the main result (Table 1). In THK-based analysis, the differences of mean cortical thickness, global THK retention, and FLUTE composite retention between subtypes were not significant which were found in the main result (Table 1). None of the neuropsychological tests were significant in these single image-based analyses.

(2) MRI and THK

Topographical map


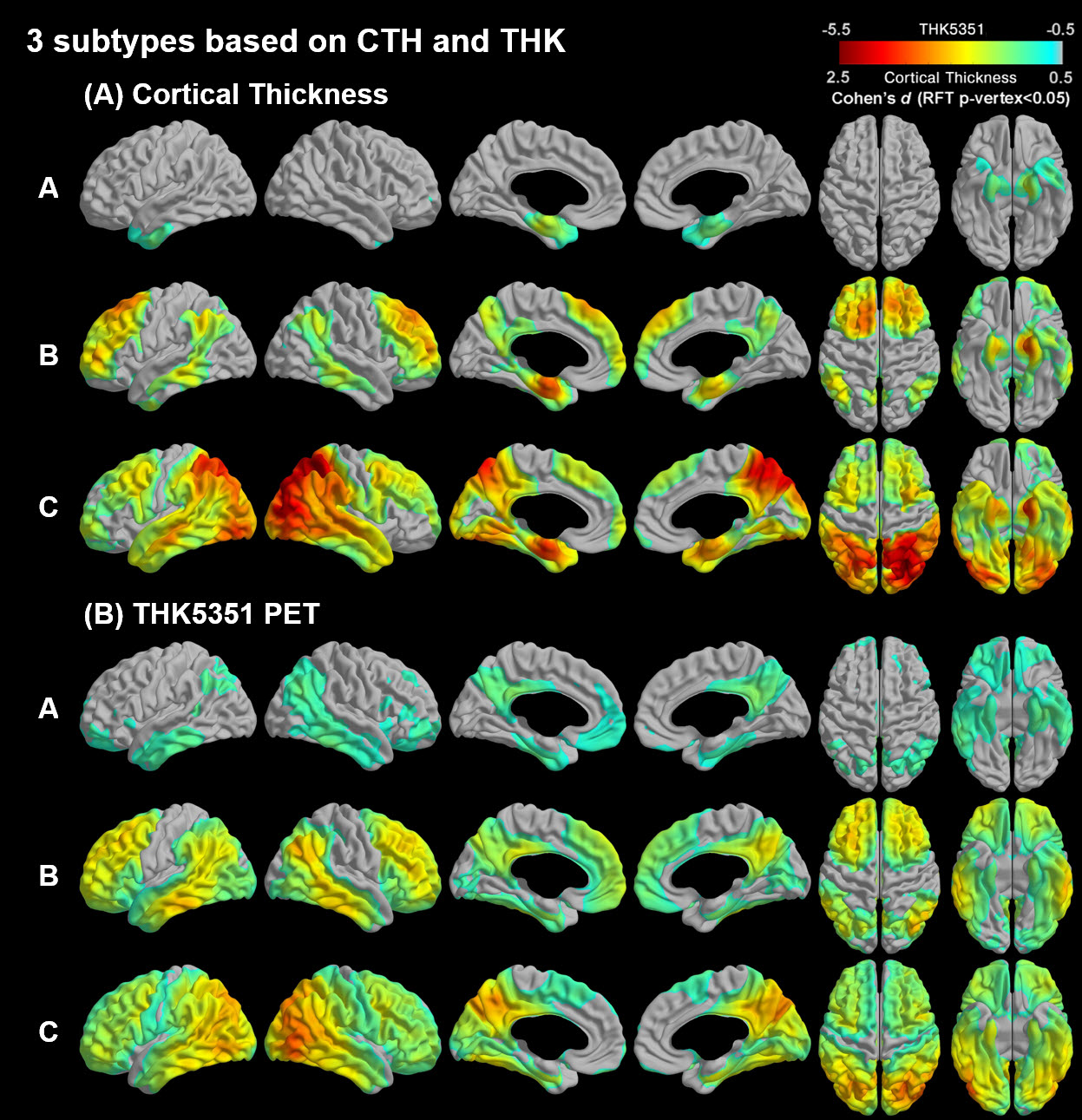


Demographic and clinical data

| **Demographic and clinical characteristics based on MRI and THK** | | | | |
| --- | --- | --- | --- | --- |
| **Variables** | **AD subtypes** | | | |
|  | **A (*n* = 41)** | **B (*n* = 22)** | **C (*n* = 20)** | ***p* value** |
| Age at scan, years | 69.66 ± 10.17 | 68.09 ± 9.93 | 62.65 ± 8.85 | 0.036*,† |
| Onset age | 66.26 ± 10.17 | 64.55 ± 9.88 | 58.99 ± 7.19 | 0.022*,† |
| Sex, female, *n* (%) | 31 (75.6%) | 15 (54.5%) | 16 (80.0%) | 0.128 |
| Education, year | 7.39 ± 4.45 | 9.45 ± 3.23 | 9.40 ± 5.56 | 0.122 |
| Disease duration, month | 40.76 ± 17.14 | 42.50 ± 25.11 | 43.90 ± 27.59 | 0.867 |
| Mean CTh, mm | 2.34 ± 0.13 | 2.31 ± 0.14 | 2.35 ± 0.15 | 0.642 |
| Global THK5351 retention | 1.62 ± 0.19 | 1.69 ± 0.19 | 1.67 ± 0.21 | 0.393 |
| Cortical composite FLUTE retention | 2.19 ± 0.37 | 2.17 ± 0.33 | 2.16 ± 0.36 | 0.940 |
| APOE4 carrier, *n* (%) | 24 (58.5%) | 10 (45.5%) | 10 (50.0%) | 0.583 |
| MMSE | 18.92 ± 5.12 | 18.50 ± 5.73 | 18.58 ± 3.89 | 0.942 |
| CDR-SOB | 4.20 ± 1.94 | 4.66 ± 1.95 | 4.15 ± 1.79 | 0.600 |
| NC, normal control; AD, Alzheimer’s disease; MT, medial temporal–dominant subtype; P, parietal-dominant subtype; D, diffuse atrophy subtype; CTh, cortical thickness; FLUTE, flutemetamol; SUVR, standardized uptake value ratio; MMSE, Mini-Mental State Examination; CDR-SOB, Clinical Dementia Rating–Sum of Boxes  One-way analysis of variance was used for comparison among AD subtypes followed by a Bonferroni post-hoc test.  Chi square test (χ^2^) was used for nominal variables.  Data are shown as mean ± standard deviation or number (%).  *Significant *p* values between groups (*p* < 0.05).  †Significant difference (*p* < 0.05) between A and C subtypes. | | | | |

Neuropsychological test results

| **Neuropsychological test results based on MRI and THK** | | | | | |
| --- | --- | --- | --- | --- | --- |
|  | | **A (*n* = 41)** | **B (*n* = 22)** | **C (*n* = 20)** | ***p* value** |
| Attention | | | | | |
|  | Digit Span Forward | -0.22 ± 1.42 | -0.12 ± 1.20 | -0.31 ± 1.12 | 0.906 |
|  | Digit Span Backward | -0.81 ± 1.26 | -1.12 ± 1.51 | -1.24 ± 1.38 | 0.479 |
| Language and related function | | | | | |
|  | K-BNT | -2.01 ± 1.96 | -1.83 ± 2.05 | -1.58 ± 2.58 | 0.781 |
| Visuospatial function | | | | | |
|  | RCFT copy | -2.10 ± 4.31 | -3.46 ± 4.97 | -4.67 ± 6.21 | 0.176 |
| Memory | | | | | |
|  | SVLT, immediate recall | -1.75 ± 1.19 | -1.85 ± 1.29 | -1.94 ± 0.78 | 0.833 |
|  | SVLT, delayed recall | -2.20 ± 0.76 | -2.23 ± 0.83 | -2.55 ± 0.76 | 0.277 |
|  | SVLT, recognition | -2.04 ± 1.42 | -2.20 ± 1.71 | -2.16 ± 1.36 | 0.919 |
|  | RCFT, immediate recall | -1.44 ± 0.81 | -1.65 ± 0.70 | -1.94 ± 0.79 | 0.083 |
|  | RCFT, delayed recall | -1.67 ± 0.95 | -2.11 ± 0.59 | -2.09 ± 0.99 | 0.104 |
|  | RCFT, recognition | -2.03 ± 1.62 | -1.84 ± 1.23 | -1.77 ± 1.34 | 0.787 |
| Frontal executive function | | | | | |
|  | COWAT, animal | -1.80 ± 0.97 | -2.04 ± 0.97 | -1.81 ± 0.95 | 0.629 |
|  | COWAT, supermarket | -1.30 ± 1.06 | -1.74 ± 0.77 | -1.64 ± 0.84 | 0.181 |
|  | COWAT, phonemic total | -1.03 ± 1.23 | -1.17 ± 1.16 | -1.12 ± 1.21 | 0.905 |
|  | Stroop test, color reading | -1.65 ± 1.26 | -1.90 ± 1.46 | -2.05 ± 1.35 | 0.556 |
|  | TMT-A | -4.37 ± 7.62 | -4.77 ± 11.86 | -3.84 ± 5.44 | 0.947 |
|  | TMT-B | -5.35 ± 5.06 | -5.56 ± 5.94 | -7.19 ± 6.37 | 0.525 |
| AD, Alzheimer’s disease; MT, Medial temporal-dominant; P, parietal-dominant; D, diffuse atrophy; K-BNT, Korean version of the Boston naming test; RCFT, Rey-Osterrieth complex figure test; SVLT, Seoul verbal learning test; COWAT, controlled oral word association test; TMT-A/B, trail making test type A/B  Data are presented as mean ± standard deviation. All data are z-scores derived on age- and education-adjusted norms.  Analysis of variance followed by Bonferroni post hoc test was used. | | | | | |

The MRI and THK-based clustering showed better topographical classification than the single image-based analyses. The brain topography of subtype A, B, and C generally matched with MT, D, and P subtypes of the main results (Figure 2). However, differences in sex, mean cortical thickness, THK global retention, and FLUTE composite retention between subtypes were not significant which were found in the main analysis. None of neuropsychological tests were significant in this MRI and THK-based analysis. This may be suggesting that the one or two image-based method without FLUTE is not enough for the subtype analysis for the early AD.

**Appendix 6. Clustering analyses with two or four subgroups based on MRI, THK5351 PET, and FLUTE PET**

(1) Dendrogram


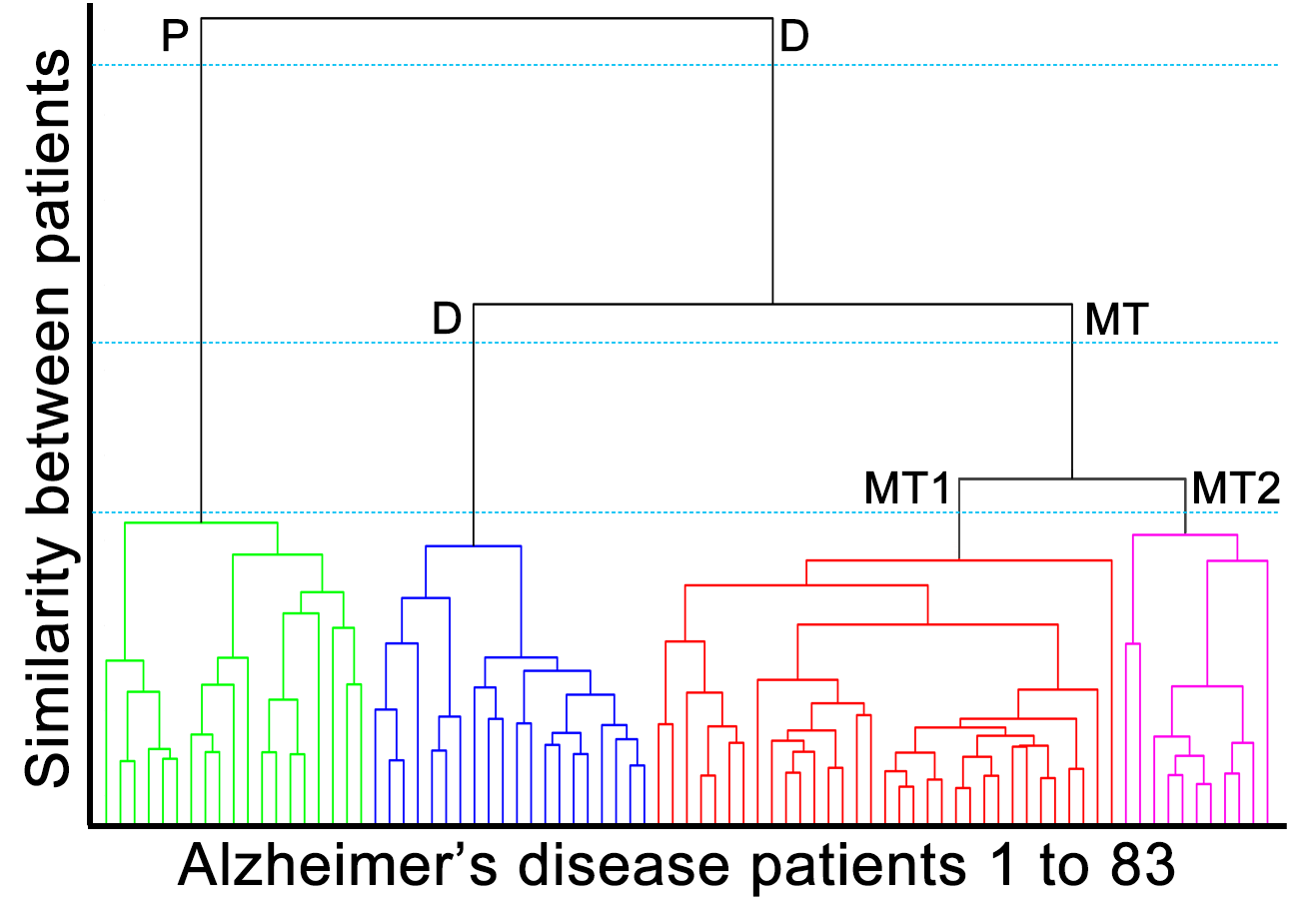


(2) Topographical map


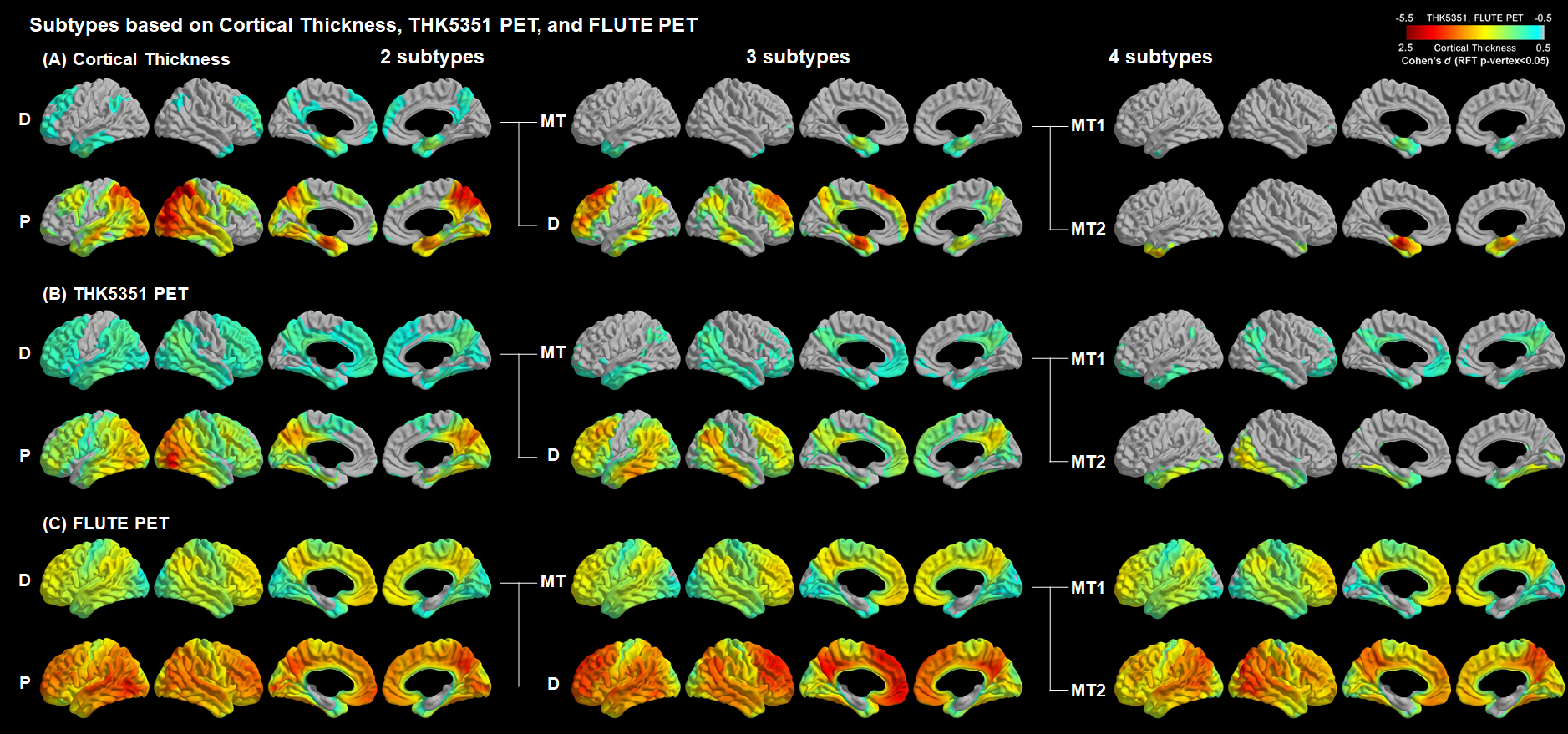


(3) Demographics, clinical data, and neuropsychological test results

Two subgroups (P and D type)

| **Demographic and clinical characteristics for AD subtypes** | | | |
| --- | --- | --- | --- |
| **Variables** | **2 subtypes** | | |
|  | **P (*n* = 19)** | **D (*n* = 64)** | ***p* value** |
| Age at scan, years | 59.47 ± 7.21 | 69.95 ± 9.61 | < 0.001* |
| Onset age | 56.12 ± 6.09 | 66.41 ± 9.48 | < 0.001* |
| Sex, female, *n* (%) | 13 (68.4%) | 46 (71.9%) | 0.771 |
| Education, year | 9.18 ± 4.74 | 8.20 ± 4.49 | 0.407 |
| Disease duration, month | 40.26 ± 20.04 | 42.48 ± 22.51 | 0.701 |
| Mean CTh, mm | 2.25 ± 0.13 | 2.36 ± 0.13 | 0.001* |
| Global THK5351 retention | 1.81 ± 0.19 | 1.60 ± 0.17 | < 0.001* |
| Cortical composite FLUTE retention | 2.39 ± 0.48 | 2.11 ± 0.28 | 0.026* |
| APOE4 carrier, *n* (%) | 7 (36.8%) | 37 (57.8%) | 0.108 |
| MMSE | 17.59 ± 4.54 | 19.03 ± 5.09 | 0.292 |
| CDR-SOB | 4.95 ± 1.74 | 4.12 ± 1.91 | 0.094 |
| NC, normal control; AD, Alzheimer’s disease; P, parietal-dominant subtype; D, diffuse atrophy subtype; CTh, cortical thickness; FLUTE, flutemetamol; SUVR, standardized uptake value ratio; MMSE, Mini-Mental State Examination; CDR-SOB, Clinical Dementia Rating–Sum of Boxes  Independent t-test was used for continuous variables and chi square test (χ^2^) was used for nominal variables.  Data are shown as mean ± standard deviation or number (%).  *Significant *p* values between groups (*p* < 0.05). | | | |

| **Neuropsychological test results for AD subtypes** | | | | |
| --- | --- | --- | --- | --- |
|  | | **P subtype**  **(*n* = 19)** | **D subtype**  **(*n* = 64)** | ***p* value** |
| Attention | |  |  |  |
|  | Digit Span Forward | -1.08 ± 1.48 | 0.04 ± 1.12 | 0.001* |
|  | Digit Span Backward | -1.94 ± 1.41 | -0.72 ± 1.21 | 0.001* |
| Language and related function | |  |  |  |
|  | K-BNT | -2.79 ± 3.05 | -1.62 ± 1.71 | 0.145 |
| Visuospatial function | |  |  |  |
|  | RCFT copy | -9.01 ± 6.40 | -1.26 ± 2.66 | <0.001* |
| Memory | |  |  |  |
|  | SVLT, immediate recall | -2.38 ± 1.23 | -1.66 ± 1.06 | 0.016* |
|  | SVLT, delayed recall | -2.48 ± 0.81 | -2.23 ± 0.77 | 0.248 |
|  | SVLT, recognition | -2.50 ± 1.46 | -2.00 ± 1.46 | 0.205 |
|  | RCFT, immediate recall | -2.10 ± 0.67 | -1.46 ± 0.78 | 0.002* |
|  | RCFT, delayed recall | -2.37 ± 0.71 | -1.73 ± 0.90 | 0.007* |
|  | RCFT, recognition | -2.44 ± 1.24 | -1.77 ± 1.49 | 0.087 |
| Frontal executive function | |  |  |  |
|  | COWAT, animal | -2.25 ± 0.70 | -1.75 ± 0.99 | 0.047* |
|  | COWAT, supermarket | -1.93 ± 0.82 | -1.37 ± 0.95 | 0.027* |
|  | COWAT, phonemic total | -1.71 ± 1.27 | -0.90 ± 1.11 | 0.010* |
|  | Stroop test, color reading | -2.90 ± 1.33 | -1.52 ± 1.18 | <0.001* |
|  | TMT-A | -11.07 ± 12.60 | -2.58 ± 6.04 | 0.018* |
|  | TMT-B | -10.09 ± 7.92 | -4.66 ± 4.15 | 0.017* |
| AD, Alzheimer’s disease; MT, Medial temporal-dominant; P, parietal-dominant; D, diffuse atrophy; K-BNT, Korean version of the Boston naming test; RCFT, Rey-Osterrieth complex figure test; SVLT, Seoul verbal learning test; COWAT, controlled oral word association test; TMT-A/B, trail making test type A/B  Independent t-test was used and data are presented as mean ± standard deviation.  All data are z-scores derived on age- and education-adjusted norms.  *Significant *p* values (*p* <0.05). | | | | |

Four subgroups (MT1, MT2, P and D type)

| **Demographic and clinical characteristics for AD subtypes** | | | | | |
| --- | --- | --- | --- | --- | --- |
| **Variables** | **4 subtypes** | | | | |
|  | **MT1 (*n* = 33)** | **MT2 (*n* = 11)** | **P (*n* = 19)** | **D (*n* = 20)** | ***p* value** |
| Age at scan, years | 70.79 ± 9.83 | 77.00 ± 6.03 | 59.47 ± 7.21 | 64.70 ± 8.07 | < 0.001*,†,[‡](https://en.wikipedia.org/wiki/Vertical_Bar),‖ |
| Onset age | 67.28 ± 9.47 | 73.16 ± 6.81 | 56.12 ± 6.09 | 61.27 ± 8.19 | < 0.001*,†,[‡](https://en.wikipedia.org/wiki/Vertical_Bar),‖ |
| Sex, female, *n* (%) | 27 (81.8%) | 10 (90.9%) | 13 (68.4%) | 9 (45%) | 0.055 |
| Education, year | 7.53 ± 4.19 | 7.45 ± 5.29 | 9.18 ± 4.74 | 9.70 ± 4.35 | 0.275 |
| Disease duration, month | 42.06 ± 23.67 | 46.09 ± 23.71 | 40.26 ± 20.04 | 41.20 ± 20.78 | 0.916 |
| Mean CTh, mm | 2.39 ± 0.10 | 2.34 ± 0.13 | 2.25 ± 0.13 | 2.31 ± 0.14 | 0.001*,† |
| Global THK5351 retention | 1.52 ± 0.12 | 1.61 ± 0.12 | 1.81 ± 0.19 | 1.74 ± 0.18 | < 0.001*,†,§,[‡](https://en.wikipedia.org/wiki/Vertical_Bar) |
| Cortical composite FLUTE retention | 2.02 ± 0.26 | 2.18 ± 0.23 | 2.39 ± 0.48 | 2.24 ± 0.29 | 0.002*,† |
| APOE4 carrier, *n* (%) | 21 (63.6%) | 7 (63.6%) | 7 (36.8%) | 9 (45.0%) | 0.212 |
| MMSE | 19.15 ± 4.38 | 20.09 ± 5.75 | 17.59 ± 4.54 | 18.21 ± 5.93 | 0.549 |
| CDR-SOB | 3.59 ± 1.54 | 4.82 ± 2.53 | 4.95 ± 1.74 | 4.60 ± 1.93 | 0.040* |
| NC, normal control; AD, Alzheimer’s disease; P, parietal-dominant subtype; D, diffuse atrophy subtype; CTh, cortical thickness; FLUTE, flutemetamol; SUVR, standardized uptake value ratio; MMSE, Mini-Mental State Examination; CDR-SOB, Clinical Dementia Rating–Sum of Boxes  One-way analysis of variance was used for comparison among AD subtypes followed by a Bonferroni post-hoc test.  Chi square test (χ^2^) was used for nominal variables.  Data are shown as mean ± standard deviation or number (%)  *Significant *p* values between groups (*p* < 0.05).  †Significant difference (*p* <0.05) between MT1 and P subtypes.  §Significant difference (*p* <0.05) between MT1 and D subtypes.  [‡](https://en.wikipedia.org/wiki/Vertical_Bar)Significant difference (*p* <0.05) between MT2 and P subtypes.  ‖Significant difference (*p* <0.05) between MT2 and D subtypes. | | | | | |

| **Neuropsychological test results for AD subtypes** | | | | | | |
| --- | --- | --- | --- | --- | --- | --- |
|  | | **MT1 subtype**  **(*n* = 33)** | **MT2 subtype**  **(*n* = 11)** | **P subtype**  **(*n* = 19)** | **D subtype**  **(*n* = 20)** | ***p* value** |
| Attention | |  |  |  |  |  |
|  | Digit Span Forward | 0.04 ± 1.17 | 0.48 ± 1.24 | -1.08 ± 1.48 | -0.22 ± 0.92 | 0.004*,†,[‡](https://en.wikipedia.org/wiki/Vertical_Bar) |
|  | Digit Span Backward | -0.40 ± 1.08 | -0.40 ± 1.11 | -1.94 ± 1.41 | -1.43 ± 1.23 | <0.001*,†,§,[‡](https://en.wikipedia.org/wiki/Vertical_Bar) |
| Language and related function | |  |  |  |  |  |
|  | K-BNT | -1.22 ± 1.15 | -2.40 ± 1.85 | -2.79 ± 3.05 | -1.82 ± 2.22 | 0.072 |
| Visuospatial function | |  |  |  |  |  |
|  | RCFT copy | -0.34 ± 1.52 | 0.06 ± 1.29 | -9.01 ± 6.40 | -3.72 ± 3.25 | <0.001*,†,§,[‡](https://en.wikipedia.org/wiki/Vertical_Bar),¶ |
| Memory | |  |  |  |  |  |
|  | SVLT, immediate recall | -1.37 ± 0.81 | -1.45 ± 0.99 | -2.38 ± 1.23 | -2.26 ± 1.25 | 0.002*,†,§ |
|  | SVLT, delayed recall | -2.23 ± 0.53 | -1.84 ± 0.78 | -2.48 ± 0.81 | -2.46 ± 1.03 | 0.125 |
|  | SVLT, recognition | -1.74 ± 1.34 | -1.81 ± 1.55 | -2.50 ± 1.46 | -2.55 ± 1.53 | 0.137 |
|  | RCFT, immediate recall | -1.33 ± 0.80 | -1.41 ± 0.73 | -2.10 ± 0.67 | -1.74 ± 0.72 | 0.005*,† |
|  | RCFT, delayed recall | -1.61 ± 0.91 | -1.66 ± 0.76 | -2.37 ± 0.71 | -1.97 ± 0.96 | 0.027*,† |
|  | RCFT, recognition | -1.57 ± 1.14 | -1.44 ± 1.31 | -2.44 ± 1.24 | -2.28 ± 1.96 | 0.090 |
| Frontal executive function | |  |  |  |  |  |
|  | COWAT, animal | -1.55 ± 0.87 | -1.41 ± 1.05 | -2.25 ± 0.70 | -2.27 ± 1.00 | 0.005*,§ |
|  | COWAT, supermarket | -1.26 ± 0.73 | -0.62 ± 1.19 | -1.93 ± 0.82 | -1.97 ± 0.76 | <0.001*,§,[‡](https://en.wikipedia.org/wiki/Vertical_Bar),‖ |
|  | COWAT, phonemic total | -0.69 ± 1.03 | -0.21 ± 0.85 | -1.71 ± 1.27 | -1.67 ± 0.98 | <0.001*,†,§,[‡](https://en.wikipedia.org/wiki/Vertical_Bar),‖ |
|  | Stroop test, color reading | -1.09 ± 0.93 | -1.09 ± 0.93 | -2.90 ± 1.33 | -2.50 ± 1.13 | <0.001*,†,§,[‡](https://en.wikipedia.org/wiki/Vertical_Bar),‖ |
|  | TMT-A | 0.77 ± 1.74 | -1.35 ± 2.66 | -11.07 ± 12.60 | -6.13 ± 9.54 | <0.001*,†,[‡](https://en.wikipedia.org/wiki/Vertical_Bar) |
|  | TMT-B | -3.95 ± 3.87 | -2.99 ± 2.37 | -10.09 ± 7.92 | -6.63 ± 4.69 | 0.001*,†,[‡](https://en.wikipedia.org/wiki/Vertical_Bar) |
| AD, Alzheimer’s disease; MT, Medial temporal-dominant; P, parietal-dominant; D, diffuse atrophy; K-BNT, Korean version of the Boston naming test; RCFT, Rey-Osterrieth complex figure test; SVLT, Seoul verbal learning test; COWAT, controlled oral word association test; TMT-A/B, trail making test type A/B  Data are presented as mean ± standard deviation. All data are z-scores derived on age- and education-adjusted norms.  Analysis of variance followed by Bonferroni post hoc test was used.  *Significant *p* values between groups (*p* < 0.05).  †Significant difference (*p* <0.05) between MT1 and P subtypes.  §Significant difference (*p* <0.05) between MT1 and D subtypes.  [‡](https://en.wikipedia.org/wiki/Vertical_Bar)Significant difference (*p* <0.05) between MT2 and P subtypes.  ‖Significant difference (*p* <0.05) between MT2 and D subtypes.  ¶Significant difference (*p* <0.05) between P and D subtypes. | | | | | | |

The results of two or four clusters based on MRI, THK5351 PET, and FLUTE PET. The main results of AD subtypes using three cluster analysis reflect that it is better than subtypes using two cluster or four cluster analyses in showing differences in clinical or imaging characeteristics

In four cluster analysis, MT subtype was ambiguously divided into two subgroups (MT1 and MT2). In general, MT1 was similar to D subtype and MT2 was similar to P subtype in the topographical map, demographics, clinical and neuropsychological test results. There were no items showing the significant difference between MT1 and MT2 subtypes.

Taken together, we found that three-cluster analysis is optimal for the classification of AD subtypes. The optimal cluster number (k=3) revealed by the Gap statistics supports our hypothesis.
